# Genomic surveillance for multidrug-resistant or hypervirulent *Klebsiella pneumoniae* among United States bloodstream isolates

**DOI:** 10.1186/s12879-022-07558-1

**Published:** 2022-07-07

**Authors:** Travis J. Kochan, Sophia H. Nozick, Rachel L. Medernach, Bettina H. Cheung, Samuel W. M. Gatesy, Marine Lebrun-Corbin, Sumitra D. Mitra, Natalia Khalatyan, Fiorella Krapp, Chao Qi, Egon A. Ozer, Alan R. Hauser

**Affiliations:** 1 Department of Microbiology-Immunology, Northwestern University, Feinberg School of Medicine, Chicago, IL USA; 2 Division of Infectious Diseases, Department of Medicine, Northwestern University, Feinberg School of Medicine, Chicago, IL USA; 3 Department of Pathology, Northwestern University, Feinberg School of Medicine, Chicago, IL USA; 4 Center for Pathogen Genomics and Microbial Evolution, Havey Institute for Global Health, Northwestern University Feinberg School of Medicine, Chicago, IL USA

**Keywords:** *Klebsiella pneumoniae*, Hypervirulent *Klebsiella*, Bacteremia, Pathogenesis, Whole-genome sequencing

## Abstract

**Background:**

*Klebsiella pneumoniae* strains have been divided into two major categories: classical *K. pneumoniae,* which are frequently multidrug-resistant and cause hospital-acquired infections in patients with impaired defenses, and hypervirulent *K. pneumoniae,* which cause severe community-acquired and disseminated infections in normal hosts. Both types of infections may lead to bacteremia and are associated with significant morbidity and mortality. The relative burden of these two types of *K. pneumoniae* among bloodstream isolates within the United States is not well understood.

**Methods:**

We evaluated consecutive *K. pneumoniae* isolates cultured from the blood of hospitalized patients at Northwestern Memorial Hospital (NMH) in Chicago, Illinois between April 2015 and April 2017. Bloodstream isolates underwent whole genome sequencing, and sequence types (STs), capsule loci (KLs), virulence genes, and antimicrobial resistance genes were identified in the genomes using the bioinformatic tools *Kleborate* and *Kaptive.* Patient demographic, comorbidity, and infection information, as well as the phenotypic antimicrobial resistance of the isolates were extracted from the electronic health record. Candidate hypervirulent isolates were tested in a murine model of pneumonia, and their plasmids were characterized using long-read sequencing. We also extracted STs, KLs, and virulence and antimicrobial resistance genes from the genomes of bloodstream isolates submitted from 33 United States institutions between 2007 and 2021 to the National Center for Biotechnology Information (NCBI) database.

**Results:**

Consecutive *K. pneumoniae* bloodstream isolates (n = 104, one per patient) from NMH consisted of 75 distinct STs and 51 unique capsule loci. The majority of these isolates (n = 58, 55.8%) were susceptible to all tested antibiotics except ampicillin, but 17 (16.3%) were multidrug-resistant. A total of 32 (30.8%) of these isolates were STs of known high-risk clones, including ST258 and ST45. In particular, 18 (17.3%) were resistant to ceftriaxone (of which 17 harbored extended-spectrum beta-lactamase genes) and 9 (8.7%) were resistant to meropenem (all of which harbored a carbapenemase genes). Four (3.8%) of the 104 isolates were hypervirulent *K. pneumoniae,* as evidenced by hypermucoviscous phenotypes, high levels of virulence in a murine model of pneumonia, and the presence of large plasmids similar to characterized hypervirulence plasmids. These isolates were cultured from patients who had not recently traveled to Asia. Two of these hypervirulent isolates belonged to the well characterized ST23 lineage and one to the re-emerging ST66 lineage. Of particular concern, two of these isolates contained plasmids with *tra* conjugation loci suggesting the potential for transmission. We also analyzed 963 publicly available genomes of *K. pneumoniae* bloodstream isolates from locations within the United States. Of these, 465 (48.3%) and 760 (78.9%) contained extended-spectrum beta-lactamase genes or carbapenemase genes, respectively, suggesting a bias towards submission of antibiotic-resistant isolates. The known multidrug-resistant high-risk clones ST258 and ST307 were the predominant sequence types. A total of 32 (3.3%) of these isolates contained aerobactin biosynthesis genes and 26 (2.7%) contained at least two genetic features of hvKP strains, suggesting elevated levels of virulence. We identified 6 (0.6%) isolates that were STs associated with hvKP: ST23 (n = 4), ST380 (n = 1), and ST65 (n = 1).

**Conclusions:**

Examination of consecutive isolates from a single center demonstrated that multidrug-resistant high-risk clones are indeed common, but a small number of hypervirulent *K. pneumoniae* isolates were also observed in patients with no recent travel history to Asia, suggesting that these isolates are undergoing community spread in the United States. A larger collection of publicly available bloodstream isolate genomes also suggested that hypervirulent *K. pneumoniae* strains are present but rare in the USA; however, this collection appears to be heavily biased towards highly antibiotic-resistant isolates (and correspondingly away from hypervirulent isolates).

**Supplementary Information:**

The online version contains supplementary material available at 10.1186/s12879-022-07558-1.

## Background

*Klebsiella pneumoniae* is a worldwide cause of bloodstream infections [1]. This bacterium can also cause severe secondary pneumonia in patients with respiratory virus infections such as SARS-CoV-2 [2]. In North America, this organism is the second leading cause of gram-negative bacteremia and is predominantly hospital-acquired or healthcare-associated rather than community acquired [3, 4]. Infections of the bloodstream are associated with higher mortality rates (20–33%) than those involving other sites [5].

Most *K. pneumoniae* infections are caused by “classical” (cKP) strains, which infect chronically ill patients residing in hospitals and long-term care facilities [6]. Some cKP strains have become increasingly resistant to antibiotics, including carbapenems [7–12]. A subgroup of cKP have been designated “high-risk clones,” lineages that are frequently multidrug-resistant (MDR) or extensively drug-resistant (XDR) and that have spread across continents to cause large numbers of infections [13, 14]. Some of the best characterized *K. pneumoniae* high-risk clones include ST11, ST14, ST15, ST17, ST147, ST45, ST258, ST307, and ST512 [15–21]. Within the United States, ST258 and ST307 are endemic high-risk clones that are usually resistant to carbapenems and extended-spectrum cephalosporins (e.g., ceftriaxione), respectively [22–25]. Genes conferring antimicrobial resistance in these strains are often carried by conjugative plasmids that allow for dissemination among cKP strains. Thus, it is not surprising that the IDSA, WHO, and the CDC have each deemed MDR *K. pneumoniae* as a serious public health priority in need of new therapeutic development [26–28].

A second type of *K. pneumoniae*, referred to as hypervirulent *K. pneumoniae* (hvKP), was first identified in Taiwan in 1986 as a common cause of pyogenic liver abscesses in young, otherwise healthy individuals living in the community [29–36]. Other than diabetes mellitus, these patients lacked risk factors commonly associated with cKP infections. While these strains are now common in Asia, little is known about their prevalence in the United States. Case reports document the presence of hvKP infections in North America, but only a few surveillance studies have been performed [35, 37–39]. hvKP strains frequently cause disseminated multi-site infections, express highly mucoid capsules, leading to a “hypermucoviscous” (hmv) colony phenotype, and are considerably more virulent in mouse models than cKP strains [40]. Their increased virulence is usually attributed to the presence of one of several large, non-conjugative “hypervirulence” plasmids [41–43]. The non-conjugative nature of these plasmids and the limitations imposed by the hyper-mucoid capsule on genetic exchange may restrict the hypervirulence phenotype to strains of a few sequence types (e.g., ST23, ST86, ST65, ST66) and capsule loci (e.g., KL1, KL2) [31, 44, 45]. Detection of hvKP is clinically important, as they may cause infections that require prolonged antibiotic therapy, tend to relapse, and frequently disseminate to remote sites [46].

**Fig. 1:**
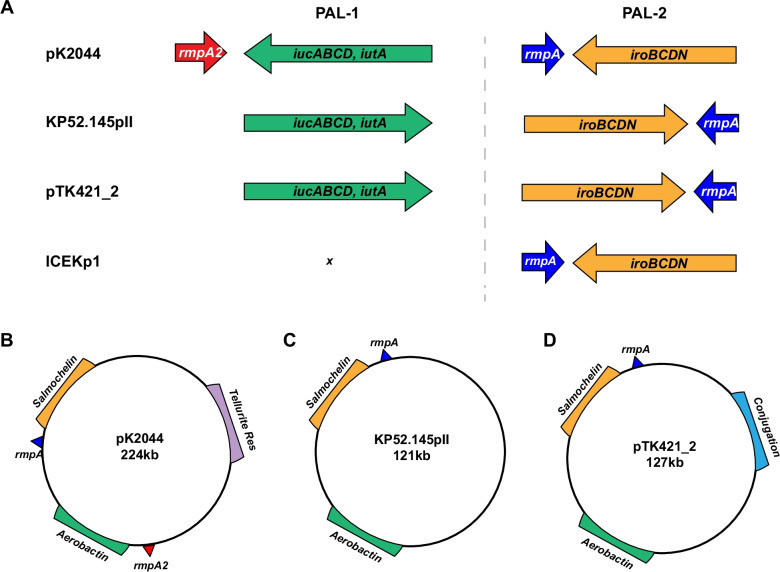
Virulence loci associated with hypervirulence in *K. pneumoniae*. PAL-1 and PAL-2 contents and organization are depicted for pK2044, KP52.145pII, pTK421_2, and ICEKp1 (**A**). Genomic elements and plasmid architecture are depicted for pK2044 (**B**), KP52.145pII (**C**) and pTK421 (**D**) (drawings not to scale)

Hypervirulence plasmids are defined by several pathogenic features. In general, these plasmids contain two distinct pathogenicity loci (PAL-1 and PAL-2) (Fig. 1A). PAL-1 usually contains a mucoid regulator gene (*rmpA2*), the aerobactin receptor gene (*iutA*), and aerobactin biosynthesis genes (*iucABCD*). PAL-2 usually consists of a distinct mucoid regulator operon (*rmpADC*), the salmochelin receptor gene (*iroN*), and salmochelin biosynthesis genes (*iroBCD*) (Fig. 1A). In addition, some of these plasmids also contain a variety of metal resistance genes including a tellurite resistance locus (*ter*), which is important for colonization of the gastrointestinal tract [47]. Considerable diversity exists among individual hypervirulence plasmids [43, 48]. For example, pK2044 and KP52.145pII, two of the most common and best characterized hypervirulence plasmids, differ in that KP52.145pII lacks *ter* and contains an incomplete PAL-1 without *rmpA2* (Fig. 1A–C). We previously identified plasmid pTK421_2 from a *K. pneumoniae* bloodstream isolate from Northwestern Memorial Hospital (NMH) in Chicago, Illinois (Fig. 1D) [49]. This plasmid also lacks *rmpA2* and *ter* but contains conjugation genes (Fig. 1D). The population diversity of hypervirulence plasmids remains poorly defined but is an area of active investigation.

The hypervirulence plasmids contribute to the pathogenicity of hvKP strains in several ways. The mucoid regulators *rmpA* and *rmpA2* increase capsule production and hypermucoviscosity, and the siderophores are thought to provide access to host-sequestered iron reserves [42]. However, the relative importance of the different accessory plasmid and chromosomal siderophores (aerobactin, salmochelin, and yersiniabactin) in hvKP virulence remain controversial [50, 51]. A 2015 report suggested that aerobactin was the only siderophore required for the hypervirulence phenotype of the ST86-K2 capsule type strain hvKP1 in both mouse intraperitoneal and pneumonia models [51]. In contrast, another report showed that only a triple siderophore mutant (disruption of genes encoding yersiniabactin, aerobactin, and salmochelin) of the ST23-K1 strain NTUH-K2044 was attenuated in the mouse intraperitoneal model [50]. In addition, strain KPPR1 (a rifampin-resistant variant of ATCC43816) is hypervirulent in a mouse model of pneumonia but is otherwise atypical in that it lacks a hypervirulence plasmid but contains a chromosomal version of PAL-2 in an integrative and conjugative element (ICEKp1, Fig. 1A) [52, 53]. Yersiniabactin production is commonly associated with hvKP lineages as part of a second ICE (ICE*Kp10*) that includes genes for both yersinibactin and the genotoxin colibactin [54]. In a KPPR1 background, yersiniabactin promoted disease in the lung and salmochelin promoted dissemination to tissues [55]. Thus, mucoid regulators and siderophores play roles in conferring the hypervirulent phenotype, but significant strain-to-strain variability may occur.

As mentioned, hvKP strains were first identified based upon the unusual disease manifestations they caused (e.g., pyogenic liver abscesses), but considerable effort has been devoted to developing a microbiological definition that could be used by clinical laboratories to identify these strains. Three characteristics are commonly associated with hvKP: 1) hmv, 2) presence of hvKP pathogenicity loci, and 3) high levels of virulence in mouse models of infection [6, 29, 31, 40, 44, 46, 56–61]. Initially, hmv was used as a proxy for the hvKP phenotype. However, several reports have described hmv *K. pneumoniae* strains that lack typical hvKP pathogenicity loci and are not hypervirulent in mice [62–64]. As a result, genetic biomarkers have been used to define hvKP, but these definitions have varied from study to study [37, 39, 65]. Recently, Russo and colleagues systematically examined the accuracy of using *rmpA*, *rmpA2*, *peg-344*, *iucA*, and *iroB*, as diagnostic biomarkers for hvKP [40, 66]. In their cohort of 175 isolates, the presence of *iucA*, *iroB* or a hmv phenotype distinguished hvKP from cKP with an accuracy of 96%, 97%, and 90%, respectively.

Fortunately, hvKP strains remain susceptible to most antibiotics, but this appears to be changing. Antimicrobial-resistant hvKP infections are on the rise, likely due to the increase in hospital-acquired hvKp infections [3, 67, 68]. In a study from Asia, 13.7% of the *iuc*^+^ isolates contained carbapenemase genes, although only one also contained other hvKp genes such as *rmpA* or *rmpA2* [69]. This convergence of hvKP and MDR-cKp can occur in one of two ways: an hvKP isolate acquires an antibiotic resistance mobile element, or an MDR-cKp isolate acquires a plasmid or integrative and conjugative element containing hvKP virulence genes. The more widespread emergence of *K. pneumoniae* strains that are both antibiotic-resistant and hypervirulent would pose a serious threat to the healthcare system.

In this work, we characterized 140 consecutive bloodstream isolates of *Klebsiella* cultured from patients at NMH in Chicago, Illinois from 2015 – 2017 and determined ST, KL type, and the presence of virulence and antimicrobial resistance genes. In addition, we evaluated publicly available genomes of bloodstream isolates collected within the United States from 2007 – 2021. We identified numerous MDR cKP high-risk clones and several isolates that contained genomic, clinical, and virulence properties consistent with hvKP.

## Methods

### Bacterial isolates and growth conditions

The isolates used in this study were from consecutive blood cultures collected between April 2015 and April 2017 and were designated as *Klebsiella pnuemoniae* sequence complex (KpSC) by the NMH Clinical Microbiology Laboratory using a VITEK-2 platform [70]. For the sub-analysis of isolates determined to be *K. pneumoniae* (sensu stricto) by sequencing, only the first isolate cultured from each unique infection was included. Isolates used in this study are listed in Additional file 13: Table S1. For experiments, bacteria were grown at 37 °C in lysogeny broth (LB) or on LB agar plates.

### Antibiotic susceptibility testing

Antimicrobial susceptibility testing of NMH bloodstream islolates was done by the clinical microbiology laboratory using a VITEK-2 platform [70]. Minimum inhibitory concentrations for each antibiotic tested are listed in Additional file 13: Table S1. Clinical breakpoints for the designations of resistant, intermediate, or susceptible were based on guidelines of the Clinical and Laboratory Standards Institute [71–73]. MDR and XDR were defined as previously described [14].

### National Center for Biotechnology Information (NCBI) Sequencing Reads

*K. pneumoniae* Illumina sequencing reads were obtained from NCBI (assessed NCBI on Dec 29th, 2021) by filtering for isolation source (blood) and geographical location (USA). Reads were downloaded from NCBI using Sratoolkit v 2.11.1 and trimmed using Trimmomatic (v0.36). de novo assembly was performed using SPAdes version 3.9.1 [74, 75]. Contigs were filtered based on coverage in trimmed reads using a custom perl script. Downloaded reads (n = 963) represent genomes deposited from 33 United States institutions between 2007 and 2021.

### Whole-genome sequencing

Genomic DNA was prepared from a single colony cultured overnight at 37 °C in LB using the Maxwell 16 system (Promega Corp., Madison, WI, USA). Libraries for Illumina sequencing were prepared using either Nextera XT (Illumina, Inc., San Diego, CA) or Seqwell (Seqwell, MA, USA) library kits and sequenced on an Illumina MiSeq or NextSeq 500 instrument to generate paired-end 300 bp or 150 bp reads (Additional file 17: Table S5). Reads were trimmed using Trimmomatic (v0.36), and de novo assembly was performed using SPAdes version 3.9.1 [74, 75]. Nanopore sequencing was performed as described previously [76, 77]. Briefly, libraries were prepared from genomic DNA using the ligation sequencing kit (SQK-LSK109, Oxford Nanopore, UK). Libraries were sequenced on a MinION instrument using a FLO-MIN106 flow cell, and base calling and demultiplexing of sequence reads was performed using Guppy v3.4.5 [78]. Hybrid assembly and circularization of Nanopore and Illumina reads were performed using Flye v2.9 (Additional file 18: Table S6). Nanopore sequencing errors were corrected by aligning Illumina reads to the assembly using BWA v0.7.17 and using serial rounds of Pilon v1.23. Annotation was performed using the NCBI Prokaryotic Genome Annotation Pipeline [79].

### Hypermucoviscosity testing

The string test was performed as described previously [80]. Briefly, Isolates were grown overnight at 37 °C on LB agar. A single colony was lifted with a loop to evaluate the formation of a viscous string between the loop and the colony. A positive string test was defined as a string length ≥ 5 mm. A centrifugation assay was also used to assess hmv. Centrifugation of overnight bacterial cultures (5 mL LB) was performed at 3220×*g* for 10 min [81]. hmv-positive isolates were identified qualitatively by the persistence of turbidity.

### Tellurite resistance testing

For screening of tellurite resistance, *K. pneumoniae* isolates were grown overnight at 37 °C on LB agar plates supplemented with 3 μg/mL potassium tellurite (Sigma-Aldrich, USA).

### Phylogenetic analysis

FastTree 2 was used to generate a maximum-likelihood phylogenetic tree based on the core genome, which was defined as sequences present in 95% of the isolates [82]. SNPs were identified by aligning raw Illumina reads to the genome of reference strain NTUH-K2044 using bwa-0.7.15. kSNPv3.0 was used for phylogenetic analysis of assembled genomes when raw sequencing reads were not available. The accession numbers of previously reported strain sequences used in this analysis (KPPR1, KP52.145, SB5881, K180005, NCTC9494, NTUH-K2044, RJF999, and ED2) are listed in Additional file 19: Table S7. The phylogenetic trees were visualized and annotated using iTOL (v4) [83].

### Molecular typing and identification of virulence genes

Assembled whole genome sequences were analyzed with the bioinformatics tools *Kleborate* v2.1 and *Kaptive* v0.7.3 using default settings to evaluate multilocus sequence type, capsule locus type, antimicrobial resistance gene content, and the presence of virulence genes [44, 84]. MOB-suite was used to predict and type plasmids and identify putative replicons [85]. Plasmids identified by whole genome sequencing were aligned using BLAST Ring Image generator (BRIG) with an alignment threshold of 85% identity [86]. MASH (v2.3) was used to cluster plasmids based on Jaccard index, and Cytoscape (v3.8.2) was used to create plasmid networks [87]. Plasmid replicons were identified using PlasmidFinder v2.1 and MOB-suite [85, 88].

### Mouse studies

Anesthetized 6- to 8-week-old C57BL/6 female mice purchased from Jackson Labs were infected intranasally as described previously [64]. Briefly, sets of mice were anesthetized with Ketamine-xylazine cocktail (100 mg/kg and 20 mg/kg, respectively). Mice were then infected with different doses of *K. pneumoniae* and monitored for two weeks post-infection for pre-lethal illness. Experimenters were blinded to strains and inoculum sizes. Mice infected with each strain or dose were chosen at random. To minimize pain and distress, mice were euthanized with CO_2_ when they reached predetermined endpoints of > 20% weight loss, abnormal respiratory rate, or a hunched posture with minimal activity. 50% lethal dose (LD_50_) values were determined in R using the *drc* package [89]. Strain dosing, deaths, and total mice inoculated are included in Additional file 20: Table S8. The number of mice used were limited to the minimum number required to calculate an accurate LD_50_. No experiments were excluded from these data. All procedures were approved by and performed in accordance with the guidelines of the Northwestern University Animal Care and Use Committee (protocol IS00002172).

### Statistical analysis

Statistical analysis was performed using GraphPad Prism. Student T-test and analysis of variance (ANOVA) followed by the Bonferroni’s correction for multiple comparisons were performed for parametric variables. For non-parametric variables, the Mann-Whitney U test was used. For comparison of survival curves, the Mantel-Cox log rank test was used. Pearson correlation coefficient was used to determine statistical associations between LD_50_ values and siderophore production. Simpson’s Diversity index was calcuated in R using the package ‘vegan’.

## Results

### Microbiological characteristics of *Klebsiella* bloodstream isolates from a single United States medical center

**Fig. 2:**
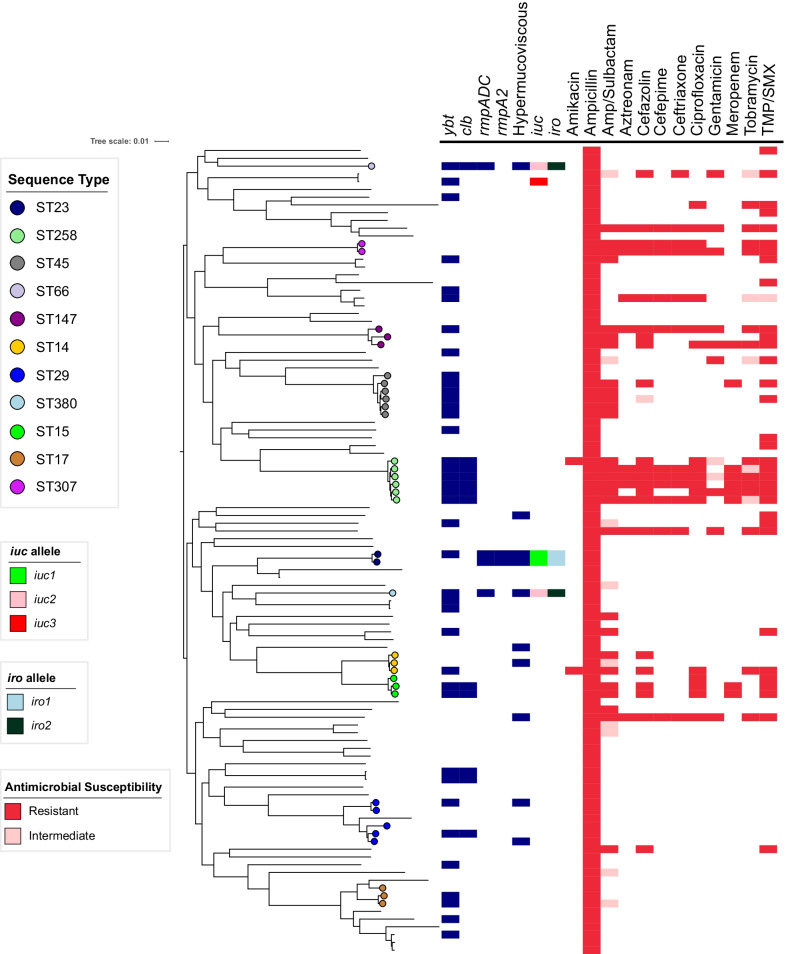
Core genome phylogenetic tree, genomic content, and antibiotic resistance of 104 K*. pneumoniae* bloodstream isolates. A Maximum likelihood phylogenetic tree was generated from core genome SNP loci in 104 K*. pneumoniae* bloodstream isolates. Sequence types, the presence of virulence genes, hypermucoviscosity, and antibiotic resistances are indicated. *ybt* = yersiniabactin biosynthesis loci, *clb* = colibactin biosynthesis loci, *rmpADC* = mucoid regulator operon, *rmpA2* = regulator of mucoid phenotype 2, *iuc* = aerobactin biosynthesis genes, *iro* = salmochelin biosynthesis genes, Amp = ampicillin, TMP/SMX = trimethoprim-sulfamethoxazole. Strains were considered hypermucoviscous if they were positive by string test. The complete genome of NTUH-K2044 was used as the reference genome

To determine the prevalence of antibiotic-resistant and hypervirulent *K. pneumoniae* strains within United States hospitals, we collected 140 consecutive bloodstream isolates identified as KpSC by the Clinical Microbiology Laboratory at NMH. These isolates were cultured from 119 patients between 2015–2017 at NMH [90]*.* Among these 140 isolates, 119 (85%) were confirmed by whole-genome sequencing to be *K. pneumoniae,* with the remaining isolates being *Klebsiella variicola* (n = 16), *Klebsiella quasipneumoniae subsp. quasipneumoniae* (n = 1), *Klebsiella quasipneumoniae subsp. similipneumoniae* (n = 3), and a non KpSC species, *Klebsiella oxytoca* (n = 1) (Additional file 1: Fig S1). The 119 *K. pneumoniae* isolates were cultured from 101 patients and consisted of 75 (STs) (Simpson’s diversity index = 0.98), 51 unique capsule loci (KL) (Simpson’s diversity index = 0.97), and 10 distinct O-antigen serotypes (Simpson’s diversity index = 0.69). After removing isolates of the same strain cultured from the same patient, we were left with 104 *K. pneumoniae* isolates that were obtained from 101 patients. Three patients had two separate *K. pneumoniae* bloodstream infections caused by distinct strains, which were included in this study. Within this collection, 32 (30.7%) isolates had STs associated with carbapenem-resistant high-risk clones such as ST258 (n = 6), ST45 (n = 6), ST29 (n = 5), ST14 (n = 3), ST17 (n = 3), ST15 (n = 3), ST147 (n = 3) (Fig. 2). Two isolates belonged to the ESBL-producing high-risk clone ST307 (Fig. 2; Additional file 2: Fig. S2A). In addition, we identified three STs associated with hvKP: ST23 (n = 2), ST66 (n = 1), and ST380 (n = 1) (Fig. 2; Additional file 2: Fig. S2A). The most common KL-types were KL2 (n = 7), KL107 (n = 7), KL30 (n = 6), KL25 (n = 5), KL24 (n = 4), KL28 (n = 4), KL22 (n = 4), and KL54 (n = 4) (Fig S2B). KL107 is associated with ST258, and KL2 is associated with ST14 and hvKP STs such as ST380 or ST66. The most common O antigen serotypes were O1 (n = 38), O2 (n = 27), and O3b (n = 21) (Additional file 2: Fig S2C). These findings indicate that bloodstream isolates at NMH are highly diverse with respect to their STs, capsule types, and O-antigen serotypes. This set of 104 *K. pneumoniae* isolates was used in the subsequent analyses.

### Antimicrobial resistance genes and alleles

We next examined the resistance profiles of the isolates in our collection of 104 bloodstream isolates to β-lactam antibiotics. Since *K. pneumoniae* is inherently resistant to ampicillin, it was not surprising that 100% of the isolates had minimal inhibitory concentrations (MICs) above the resistance threshold for this antibiotic (Fig. 2; Additional file 3: S3). Non-susceptibility rates (either resistant or intermediate) were as follows: 39 (37.5%) isolates to ampicillin/sulbactam, 17 (16.3%) to aztreonam, 23 (22.1%) to cefazolin, 18 (17.3%) to ceftriaxone, 17 (16.3%) to cefepime, and 9 (8.7%) to meropenem (Additional file 13: Table S1; Fig. 2; Additional file 3: S3). Of the 104 isolates, 44 (42%) contained at least one acquired antimicrobial resistance gene (Additional file 13: Table S1). Twelve isolates (11.5%) contained extended-spectrum beta-lactamase genes, the most common of which were *bla*_CTX-M-15_ (n = 7, 6.7%) and *bla*_SHV-12_ (n = 4, 3.8%) (Additional file 13: Table S1; Additional file 4: Fig S4). Strains carrying *bla*_CTX-M-15_ were highly resistant to antibiotics, including all beta-lactams tested except meropenem (Additional file 4: Fig S4A-F). Strains carrying *bla*_SHV-12_ also carried genes predicted to confer carbapenem resistance (Fig. 2; Additional file 4: S4A–F). Nine (8.7%) isolates contained a carbapenemase gene (8 *bla*_*KPC-3*_, 1 *bla*_*NDM-1*_) (Additional file 4: Fig S4). *bla*_*NDM-1*_ was found in an ST147 isolate, while *bla*_*KPC-3*_ carbapenemase genes were found in ST258 (n = 5), ST15 (n = 2), and ST45 (n = 1) isolates (Additional file 13: Table S1). One ST258 isolate, KPN88, did not contain a carbapenemase gene and was sensitive to several beta-lactams (Additional file 13: Table S1; Fig. 2). Four of the NMH bloodstream isolates contained both ESBL and carbapenamase genes (Fig. 2; Additional file 13: Table S1). Isolates containing one or both of these genes also carried genes predicted to confer resistance to aminoglycosides, trimethoprim, sulfonamides, tetracycline, fluoroquinolones, and phenicols, suggesting these strains contain one or more antimicrobial resistance plasmids (Additional file 13: Table S1).

Resistance to other antibiotics was also quite common in the overall collection. Thirty-four isolates (32.6%) were non-susceptible to the combination of trimethoprim and sulfamethoxazole (TMP/SMX) (Additional file 3: Fig. S3). Of these isolates 31 (29.8%) contained both the *dfrA* and *sul1/2/3* genes, which encode resistance to TMP/SMX (Additional file 13: Table S1). Two resistant isolates contained only *dfrA* and one contained only *sul2* (Additional file 13: Table S1). Eighteen isolates (17.3%) were resistant to ciprofloxacin (Additional file 3: Fig S3). Each of these isolates contained either the *qnrB* gene or a mutation in the *gyrA* gene (Additional file 13: Table S1). Non-susceptibility rates to tobramycin, gentamicin, and amikacin were 17.3%, 10.5%, and 1.9%, respectively (Additional file 3: Fig S3). The overall prevalence of aminoglycoside modifying enzyme (AME) genes was 31.7% (n = 33) (Additional file 13: Table S1). The most common AMEs were streptomycin resistance genes *strA* and *strB* (n = 22) and the streptomycin/spectinomycin adenyltransferase *aadA2* (n = 10); however, isolates containing only these AMEs were sensitive to tobramycin, gentamicin, and amikacin (Additional file 13: Table S1). The acetyltransferases *aac(6')-Ib-cr.V2* (n = 7), *aac(3)-IId* (n = 2), and *aac(3)-IIa* (n = 2), and the phosphotransferases *aph(3)-Ia.v1 (n* = *5) and aph(3’)-Ia (n* = *2)* were associated with nonsusceptibility to gentamicin and tobramycin (Additional file 13: Table S1). Collectively, 17 (16.3%) of the 104 isolates were MDR, including 12 (11.5%) that were XDR (Fig. 2). These findings indicate the MDR bacteria comprise a substantial proportion of *K. pneumoniae* strains causing bloodstream infections at NMH and suggest that many strains carry mobile elements with multiple resistance genes.

### Antimicrobial resistance plasmids

**Fig. 3:**
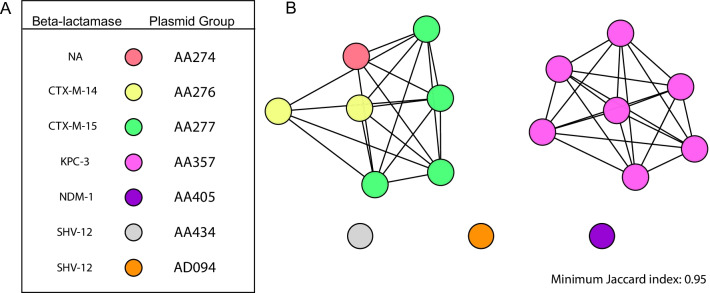
Prediction and analysis of antimicrobial-resistant plasmids in ceftriaxone-resistant *K. pneumoniae* isolates. MOB-suite was used to infer the presence and sequence of plasmids containing ESBL and carbapenemase genes in ceftriaxone-resistant isolates. Inferred plasmids were then assigned to plasmid groups (represented by different colors) based on replicon family, relaxase type, and matepair formation and transferability (**A**). Clustering of inferred plasmids was performed using Mash. Each node represents a plasmid, and two plasmids are connected by an edge if their Jaccard index is ≥ 0.95 (**B**). Networks were graphed using Cytoscape

Next, we sought to characterize the ESBL or carbapenamase plasmids harbored by the 18 NMH isolates resistant to ceftriaxone. To define plasmid sequences, we used MOB-suite to predict and reconstruct plasmid sequences in silico from short-read draft genome assemblies. We presumptively identified 16 plasmids that contained ESBL (n = 8) or carbapenamase genes (n = 8) (Additional file 14: Table S2). These 16 plasmids were predicted to belong to 8 plasmid groups that together formed a plasmid network of 5 clusters based on genomic similarity, replicon family, and antimicrobial resistance gene content (Fig. 3A; Additional file 14: Table S2). One plasmid, from strain KPN103, clustered with ESBL containing plasmids, but did not contain an ESBL (Fig. 3; Additional file 14: Table S2). To clarify the identity of these plasmid clusters, we performed Nanopore long-read sequencing on four of the ceftriaxone-resistant isolates: KPN94, KPN103, KPN107, and KPN132. These four isolates cumulatively contained five plasmids with either ESBL (n = 3) or carbapenamase (n = 2) genes (Additional file 15: Table S3). Each of these plasmids belonged to a distinct MOB cluster (Additional file 15: Table S3). While inferred plasmid content was accurate, specific clustering of the plasmids based on hybrid assemblies did not correlate well with those inferred by MOB-suite (Additional file 14: Table S2, Additional file 15: Table S3). This is likely due to the extensive plasmid diversity in *K. pneumoniae* and the tendency of these plasmids to recombine [45, 91]. These results suggest that ESBL and carbapenemase genes are carried by a variety of plasmids in our collection.

### Virulence traits

We next searched the *K. pneumoniae* genomes for genes associated with enhanced virulence. The siderophore yersiniabactin promotes pulmonary infections in animal models, is associated with more severe disease in human infections, and is produced by both hvKP and cKP strains [52, 54, 55, 92]. We identified yersiniabactin biosynthesis loci (*ybt*) in 37.5% (n = 39) of the 104 NMH bloodstream isolates (Fig. 2; Table 1). Of these 39 isolates, 10 (26%) contained ESBL or carbapenamase genes. Eleven distinct variants of *ybt* were detected, the most common being *ybt10* (n = 12), *ybt17* (n = 11) and *ybt4* (n = 4) (Additional file 13: Table S1). Although yersiniabactin biosynthetic genes are carried by several different integrative conjugative elements (ICEs), *ybt* and colibactin (*clb*) biosynthesis loci are found together on ICE*Kp10* [31]. We identified 12 (11.5%) isolates representing five STs (ST258, ST29, ST66, ST15, and ST234) that contained ICE*Kp10* (Additional file 13: Table S1). Although ICE*Kp10* elements containing *ybt*1 and *clb*2 lineages are found in the globally distributed CG23-I sub-lineage, our isolates primarily contained ICE*Kp10* elements with *ybt*17 and *clb*3, which are found in the epidemic ST258 lineage [31, 54, 93]. Indeed, in our collection 6 (50%) of the 12 isolates with ICE*Kp10* were ST258, and 7 (58%) contained ESBL or carbapenemase genes (Additional file 13: Table S1). Only one potential hvKP isolate (ST66) contained ICE*Kp10* (Additional file 13: Table S1). These findings suggest that yersiniabactin and colibactin genes were predominantly associated with cKP isolates in our collection.

**Table 1:** Virulence factors identified in U.S bloodstream isolates

NMH	Positive
Isolates n = 104	n(%)
*iroB*	4 (3.8)
*iucA*	5 (4.8)
*rmpA*	4 (3.8)
*rmpA2*	2 (1.9)
*ybt*	39 (37.5)
*clb*	12 (11.5)
*iroB/iucA/rmpA*	4 (3.8)
String Test	10 (9.6)
Centrifugation Test	18 (17.3)
Tellurite Resistance	5 (4.8)

NCBI	Positive
Isolates n = 963	n(%)
*iroB*	9 (0.9)
*iucA*	32 (3.3)
*rmpA*	14 (1.5)
*rmpA2*	21 (2.2)
*ybt*	345 (35.8)
*clb*	194 (20.1)
*iucA and rmpA/2*	25 (2.6)
CR* and *iucA*	25 (2.6)
CR* *iucA and rmpA/2*	18 (1.9)

CR* carbapenem resistant

In addition to *ybt* and *clb*, we screened our collection for the following hvKP associated factors: *iuc, iro, rmpA,* and *rmpA2*. *iuc* was identified in 5 (4.8%), *iro* in 4 (3.8%), *rmpA* in 4 (3.8%), and *rmpA2* in 2 (1.9%) of the isolates (Fig. 2; Table 1). These virulence factors tended to be clustered within the same few isolates. For example, four isolates contained *iuc, iro,* and *rmpA* (Table 2)*.* Two of these four (KPN8 and KPN115) were ST23 and the other two (KPN49 and KPN165) were ST66 and ST380, respectively. A single isolate, KPN23, was ST881 and contained *iuc* but not *iro* or *rmpA*. (Fig. 2; Table 2). Three distinct lineage variants of the *iuc* locus were identified: *iuc1* (n = 2, ST23), *iuc2* (n = 2, ST66 and ST380), and *iuc3* (n = 1, ST881) (Table 2). Although the *rmpA2* gene was found in two strains (KPN8 and KPN115), both genes contained a frameshift mutation in a previously identified homopolymer region [94]. All *iuc*^+^ isolates were sensitive to every antibiotic tested except ampicillin (Additional file 13: Table S1), consistent with the highly susceptible nature of hvKP strains.

**Table 2:** Genomic Characteristics of bloodstream isolates containing hvKp pathogenicity loci

Strain	ST	K-Locus	*iuc*	*iro*	hmv^a^	*rmpA/2*	Plasmid	Virulence Plasmid Replicon	Other Plasmid Replicon	LOGLD50
KPN8	ST23	KL1	*iuc 1*	*iro 1*	+	*rmpA, rmpA2*	pKPN8_1	repB, IncHI1B(pNDM-MAR)		2.22
KPN23	ST881	KL2	*iuc 3*	*−*	*−*	*−*	pKPN23_1	IncFIB(K), IncFII(pKP91)	ColRNAI	6.07
KPN49	ST66	KL2	*iuc 2*	*iro 2*	+	*rmpA*	pKPN49_1	IncFIB(K)	IncFIA(HI1)	1.51
KPN115	ST23	KL1	*iuc 1*	*iro 1*	+	*rmpA, rmpA2*	pK2044	repB, IncHI1B(pNDM-MAR)	IncFIB(pKPHS1)	NT^b^
KPN165	ST380	KL2	*iuc 2*	*iro 2*	+	*rmpA*	pKPN165_1	IncFIB(K)		NT^b^
KPN80	ST29	KL30	*−*	*−*	+	*−*	NT			8.06
TK421	ST34	KL20	iuc 2	iro 2	+	rmpA	pTK421_2	repB, IncR	IncFII(pKP91), IncFIA(HI1)	4.52
hvKP5	ST23	KL1	*iuc 1*	*iro 1*	+	*rmpA, rmpA2*	pK2044	repB, IncHI1B(pNDM-MAR)	IncFIB(pKPHS1)	1.98

^a^hmv: hypermucoviscosity

^b^NT: not tested

We next screened the collection for hmv by both the string test and a centrifugation assay. A total of 10 (9.6%) and 18 (17.3%) isolates, respectively, were positive for hmv by these two tests (Table 1), including all four of the isolates containing *iuc, iro,* and *rmpA* (KPN8, KPN49, KPN115, KPN165) but not the isolate containing only *iuc* (KPN23) (Table 2; Additional file 13: Table S1). These data confirm previous reports that the string test identifies fewer strains as hmv than centrifugation [64, 81]. Several isolates (e.g., KPN80) were hmv but lacked *iuc, iro, rmpA,* and *rmpA2* (Table 2). These findings are consistent with published studies showing that *rmpA* and *rmpA2* are not necessary for hmv [40, 93].

Isolates were also tested for tellurite resistance, a phenotype commonly encoded by pK2044 hypervirulence plasmids (Fig. 1). Five isolates (4.8%) grew on tellurite, including the two hmv ST23 isolates that contained *rmpA, rmpA2*, *iro1* and *iuc1* (Table 1). These results suggest that these two isolates contained pK2044-like plasmids. In contrast, the two isolates containing *iro2, iuc2* and *rmpA* were susceptible to tellurite, suggesting that they contain KP52.145pII-like plasmids. Collectively, these findings indicate that clinical isolates with genomic features of hvKP were present at NMH.

### Analysis of virulence plasmid content

To identify potential virulence plasmids, we used MOB-suite to predict plasmids from Illumina-generated sequences of all 104 isolates. This analysis identified the presence of 397 potential plasmids (Additional file 14: Table S2). Five isolates were predicted to harbor plasmids containing *iuc* (four of these also contained *iro* and *rmpA*) and five were predicted to harbor plasmids containing *ybt* (Additional file 14: Table S2). These predicted plasmids were from four distinct groups that formed four different plasmid network clusters based on replicon family, relaxase type, and matepair formation and transferability (Additional file 5: Fig S5; Additional file 14: Table S2). No other plasmids from our collection clustered with these plasmids. These data suggest that three distinct types of *iuc*^+^ plasmids and one type of *ybt* plasmid were present in our collection.

**Fig. 4:**
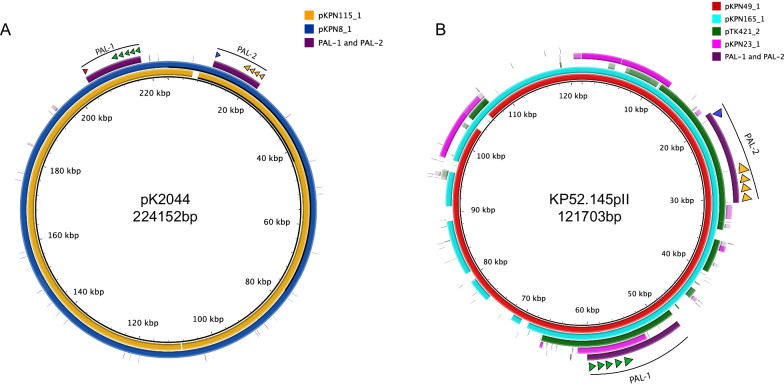
The bloodstream isolates KPN115 and KPN8 harbor pK2044-like plasmids. pKPN115_1 and pKPN8_1 sequences were aligned to the pK2044 sequence (NC_006625.1) using blast ring image generator (BRIG) (**A**). The indicated plasmids sequences were aligned to KP52.145pII using BRIG (**B**). A sequence identity threshold of 85% was used. Aerobactin biosynthesis genes are indicated with green arrows, salmochelin with orange arrows, *rmpA* with a blue arrow, and *rmpA2* with a red arrow

**Fig. 5:**
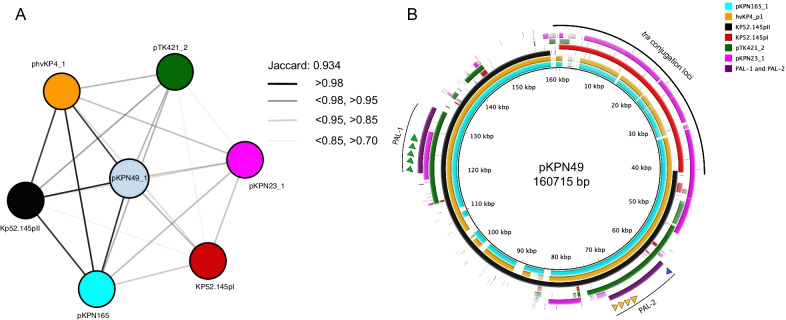
The bloodstream isolates KPN49 and KPN165 harbor KP52.145pII-like plasmids. De novo clustering of plasmids using Mash (**A**). Individual nodes represent a sequenced plasmid. The thickness of the edge connecting plasmids is determined by Jaccard index as indicated. Networks were graphed using Cytoscape. The indicated plasmid sequences were aligned to pKPN49_1 using BRIG (**B**). A sequence identity threshold of 85% was used. The ~ 40 kb *tra* locus insertion is labeled. Aerobactin biosynthesis genes are indicated with green arrows, salmochelin with orange arrows, and *rmpA* with a blue arrow. Plasmid accession numbers for previously published plasmids are listed in Table S7

We next focused on the *iuc* + plasmids. We performed Nanopore sequencing on the isolates that were predicted to harbor them, which allowed complete plasmid sequences to be generated. KPN8 and KPN115 contained plasmids pKPN8_1 and pKPN115_1, which had near complete alignment with pK2044, including plasmid replicons *repB* and *IncHI1B* and pathogenicity loci PAL-1 and PAL-2 (Fig. 4A; Table 2). A third isolate, KPN165, harbored pKPN165_1, an *IncFIB(K)* plasmid with close alignments with KP52.145pII but not pK2044 (Fig. 4B; Additional file 6: Fig. S6). Similar to KP52.145pII, this plasmid contained a version of PAL-1 with *iucABCD* and *iutA* but lacking *rmpA2* (Figs. 1, 4B). Closer examination, however, revealed that pKPN165_1 contained a ~ 40 kB insertion (absent in KP52.145pII) consisting of a *tra* conjugation locus similar to the *tra* locus of KP52.145pI, a second (non-virulence) plasmid harbored by KP52.145 (Fig. 5B) [48, 95]. We also identified a similar chimeric hypervirulence plasmid (99.0% identity) in the previously published ST380 hypervirulent strain hvKP4 (Fig. 5B) [96, 97]. The fourth isolate, KPN49, contained a plasmid pKPN49_1, which also had an *IncFIB(K)* replicon and similarity to KP52.145pII and a ~ 40-kb insert of *tra* genes (Figs. 4B, 5B). Interestingly, this isolate contained a second plasmid, pKPN49_2, with similarity to KP52.145pI and that may have served as the source of the ~ 40 kb *tra* insertion in pKPN49_1. Consistent with these alignments, pKPN49_1, pKPN165_1, KP52.145pII, and the plasmid from hvKP4 group closely with each other based on genomic similarity (Jaccard index > 0.98) (Fig. 5A). The fifth isolate, KPN23, carried pKPN23_1, a two-replicon plasmid (*IncFIB*(*K*) and *IncFII*(*pKP91*)) with conjugation genes, *iucABCD,* and *iutA* but lacking *rmpA, rmpA2,* and *iro* (Table 2). This plasmid did not resemble known hypervirulence plasmids but was highly similar to a plasmid harbored by a swine isolate KPCTRSRTH01_p2 collected in Thailand in 2016 (Additional file 7: Fig S7; Table 2). For comparison, we also included in our analysis pTK421_2, a unique hypervirulence plasmid we had previously identified from a bloodstream isolate collected at NMH in 2013 [49]. pTK421_2 contains PAL-1, PAL-2, and conjugation genes (Fig. 1). This plasmid was distinct from pK2044, KP52.145pII, and pKPN23_1 (Fig. 4B; Additional file 6: S6).

Overall, among the 104 K*. pneumoniae* bloodstream isolates we identified four isolates that contained two distinct hypervirulence plasmids, a fifth isolate that contained a plasmid containing aerobactin biosynthetic genes, and five additional isolates containing presumptive yersiniabactin plasmids.

### Phylogenetic analysis of bloodstream isolates containing hvKP pathogenicity loci

To examine the phylogenetic relationships of the five NMH bloodstream isolates containing aerobactin biosynthesis genes, we generated a core genome phylogenetic tree of these isolates along with previously published hvKP isolates. These results confirmed previous findings that KL1 hvKP isolates tend to be phylogenetically clustered, whereas KL2 hvKP isolates tend to be more dispersed (Additional file 8: Fig S8). While KPN115 and KPN8 are both ST23 isolates, they are not part of a previously described and globally distributed CG23-I clade and, as such, neither contained the combination of yersiniabactin and colibactin biosynthetic genes observed in ICE*Kp10* (Additional file 8: Fig S8) [31]. KPN49 is part of a rarely described ST66-K2 lineage that has recently re-emerged around the globe (Additional file 8: Fig S8) [95, 98, 99]. KPN165 is phylogenetically close to hvKP4, and both isolates harbor similar KP52.145pII-like plasmids containing additional putative conjugation loci. However, KPN49 harbors a very similar plasmid (99.6% identity) despite being quite divergent genetically (Additional file 8: Fig S8; Table 2). These findings suggest that this plasmid has spread by horizontal transmission, perhaps aided by the presence of the insertion containing *tra* genes. Together, these results suggest that multiple lineages with features of hvKP are present at NMH.

### Virulence in a murine model of pneumonia

**Fig. 6:**
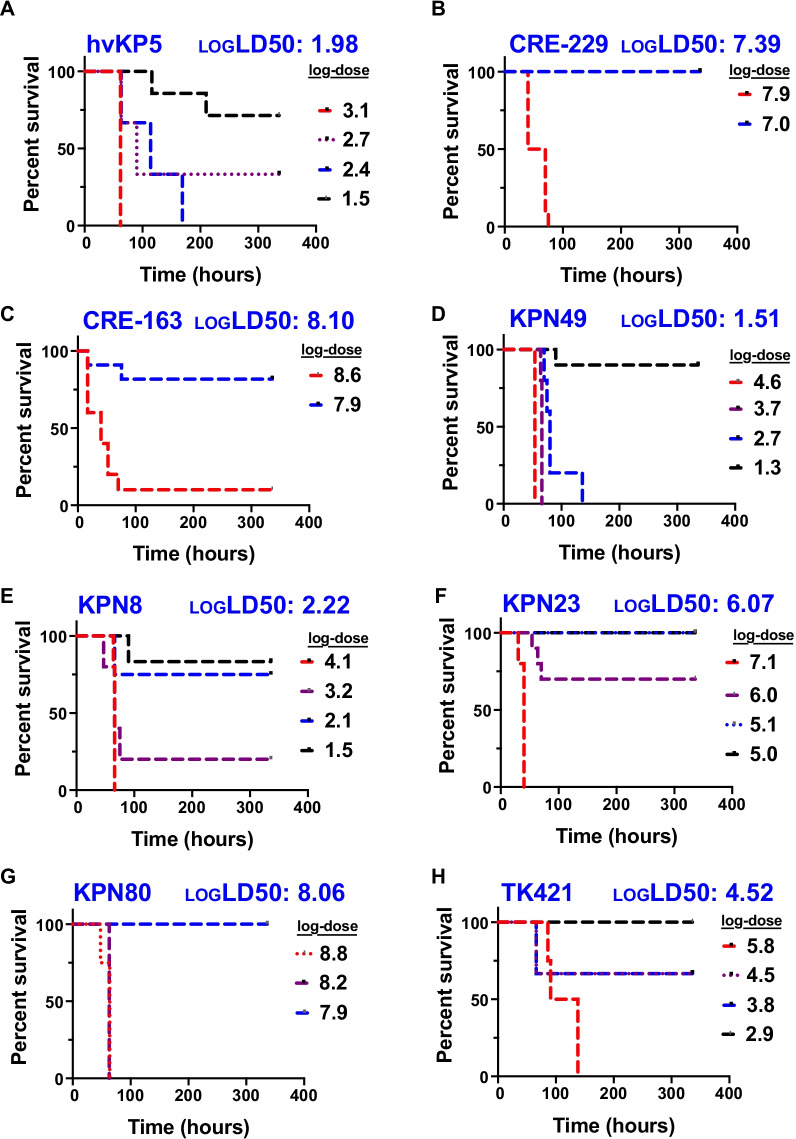
Virulence of representative NMH *K. pneumoniae* isolates containing hvKP pathogenicity loci in a murine model of pneumonia. C57BL/6 mice were infected by nasal aspiration with the indicated doses of hvKP5 (**A**), CRE-229 (**B**), CRE-163 (**C**), KPN49 (**D**), KPN8 (**E**), KPN23 (**F**), KPN80 (**G**), and TK421 (**H**). The estimated log_10_ LD_50_ values are listed in blue. The number of mice used for each dose are listed in Additional file 20: Table S8

Studies to date suggest that hvKP strains share the common feature of being highly virulent in mouse models of infection [40, 51, 97, 100]. To more definitively determine whether the five bloodstream isolates with features of hvKP were indeed hypervirulent, we measured the virulence of representative isolates in a mouse pneumonia model. To determine the level of virulence of the isolates containing characteristics of hvKP, LD_50_ measurements were made. For reference, we first determined the virulence of a published hvKP isolate, hvKP5, which is an ST23 KL1 strain that harbors pK2044 (*iuc1, iro1, rmpA,* a disrupted allele of *rmpA2*) and is hmv (Table 2; Additional file 8: Fig S8) [60]. As expected, hvKP5 was extremely virulent and had a LD_50_ of 10^2.0^ CFU (Fig. 6A). hvKP5 was slightly more virulent than previously published hvKP isolates hvKP1 and hvKP4, which have an LD_50_ of 10^3.2^ and 10^4.6^ CFU, respectively [96]. As additional controls, we tested classical isolates CRE-229 (KL107, ST258) and CRE-163 (KL112, ST15) from our previously published collection of carbapenem-resistant isolates [101]. CRE-229 and CRE-163 had LD_50s_ of 10^7.4^ and 10^8.1^ CFU, respectively (Fig. 6B, C). From the NMH collection, we selected KPN49 and KPN8 as representative pK2044 and KP52.145pII isolates, respectively. These isolates had LD_50_ values of 10^1.5^ and 10^2.2^ CFU, respectively, indicating high levels of virulence (Fig. 6D, E). In comparison, KPN23, which is non-hmv and has an *iuc3*-containing plasmid lacking other hvKP virulence genes, had an intermediate LD_50_ value of 10^6.1^ CFU (Fig. 6F). Next, we tested KPN80, an hmv bloodstream isolate from our collection lacking *iuc, iro, rmpA,* and *rmpA2* (Table 2). The hmv phenotype of KPN80 is likely due to missense mutations identified in its *wzc* gene (G132D, L147I, N248D, L607I, S629F), a part of the capsule biosynthesis loci [102]. This isolate had an LD_50_ value of 10^8.1^ CFU which is consistent with our cKP controls and previously published LD_50_ values for cKP (Fig. 6G) [96]. We also included in the analysis TK421, the previously identified hmv^+^ hvKP-like strain that contained *iuc2, iro2,* and *rmpA* (Table 2). TK421 had an LD_50_ of 10^4.5^ CFU (Fig. 6H). Collectively, these data confirm that KPN8, KPN49, and TK421 are hvKP strains and suggest that KPN115 and KPN165 are also hvKP. KPN23 has an intermediate LD_50_ value between that of hvKP and cKP.

### Siderophore production

hvKP virulence has been attributed to the secretion of unusually large amounts of siderophores [59]. For this reason, we quantified siderophore concentrations in bacterial supernatants using a chrome-azurol S (CAS) assay. We again used a set of representative isolates for these assays. Cell-free supernatants from cultures of *iuc*^+^ isolates (KPN8, KPN23, KPN49, TK421, and hvKP5) had the greatest iron chelation activity, equivalent to > 150 mM dipyridyl (Additional file 9: Fig S9A). CRE-229 and KPN80 produced somewhat less activity (> 100 mM dipyridyl equivalent), while CRE-163 produced ~ 50 mM dipyridyl equivalent (Additional file 9: Fig S9A). In these select isolates, there was a strong inverse correlation between siderophore production and log(LD_50_) (Pearson correlation coefficient − 0.86, R^2^ 0.76) (Additional file 9: Fig S9). These findings suggest that the amount of siderophore secretion is associated with *K. pneumoniae* virulence.

### Infections caused by hvKP strains

Since KPN8, KPN49, KPN115, and KPN165 had microbiological, genetic, and virulence properties of hvKP, we next examined the clinical context of the infections they caused. None of the four patients infected by these strains had a known travel history to areas where hvKP is endemic. Three of the four patients had infections that were community-acquired (Table 3). Two patients had hepatic abscesses, and one had a lung abscess. Two patients had diabetes mellitus, a known risk factor for hvKP infection [103–105]. None of the patients had other common manifestations of hvKP infections, such as endophthalmitis, meningitis, or necrotizing fasciitis. We also looked at the infections cause by TK421, KPN23, and KPN80. The patient infected with TK421, a slightly less virulent strain with features of hvKP, had a history of diabetes mellitus and presented with a community-acquired liver abscess, although this patient had predisposing conditions including a previous Whipple procedure and a history of prior liver abscesses. The patient infected with KPN23, the *iuc3*^+^ , non-hmv strain with intermediate levels of virulence, did not have features of or risk factors for hvKP infection. Likewise, the patient infected with KPN80, the hmv isolate that lacked hvKP-associated genes, also did not have features of or risk factors for hvKP infection. Although the very small number of patients prevents conclusions from being drawn, the characteristics of the infections caused by KPN8, KPN49, KPN115, and KPN165 are consistent with their classification as hvKP.

**Table 3:** Clinical Characteristics of bloodstream isolates containing hvKp pathogenicity loci

Strain	Age (years)	Type of infection	Community-acquired infection	Medical comorbidities
KPN8	67	Bloodstream only	No	Type II diabetes, myocardial infarction, peripheral vascular disease
KPN23	50	Bloodstream only	No	Pancreatic adenocarcinoma
KPN49	53	Pneumonia/lung abscess	Yes	HIV
KPN115	73	Hepatic abscess	Yes	Type II diabetes, colon adenocarcinoma
KPN165	50	Hepatic abscess	Yes	Cholangiocarcinoma
KPN80	68	Pneumonia	Yes	Cirrhosis
TK421	65	Hepatic abscess	Yes	Type II diabetes, pancreatic adenocarcinoma

### Genomic analysis of bloodstream isolates from the United States

**Fig. 7:**
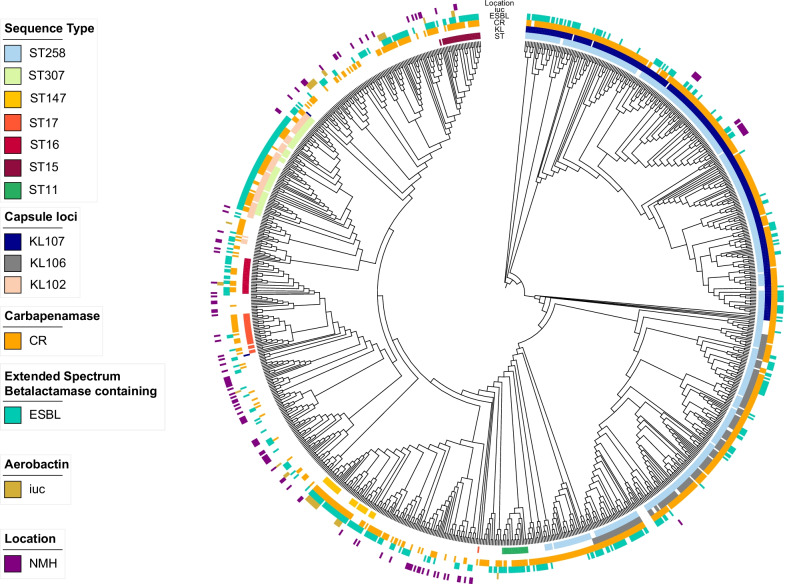
Phylogenetic tree of United States *K. pneumoniae* bloodstream isolates. Maximum likelihood phylogenetic tree generated from core genome SNP loci in 1067 *K. pneumoniae* bloodstream isolates (NMH and NCBI). Sequence types, capsule loci, and the presence of virulence and antibiotic resistance genes are indicated. *iuc* = aerobactin biosynthesis genes, CR = contains a carbapenemase, ESBL = contains an extended-spectrum beta-lactamase gene, NMH = isolate was collected from Northwestern Memorial Hospital. The complete genome of NTUH-K2044 was used as the reference genome. Branch lengths are not to scale

We next sought to examine a larger set of *K. pneumoniae* bloodstream isolates from more diverse sites across the United States for evidence of hvKP strains. For this purpose, we downloaded Illumina sequencing reads for all available bloodstream *K. pneumoniae* genomes (n = 963) from the NCBI database. Among these 963 sequences, we identified 139 unique STs, 94 unique KL, and 10 unique O-antigen serotypes (Fig. 7; Additional file 10: Fig. S10). The most common STs were endemic high-risk clones ST258 (n = 499) and ST307 (n = 63) (Additional file 10: Fig S10A, Additional file 16: Table S4). STs associated with hvKP were detected at a low prevalence (n = 6, 0.62%): ST23 (n = 4, 0.42%), ST380 (n = 1, 0.10%), and ST65 (n = 1, 0.10%). The most common KL-types were KL107 (n = 299), KL106 (n = 159), and KL102 (n = 69). KL106 and KL107 are associated with ST258, and KL102 is associated with ST307. The most common O antigen serotype was O2 (n = 640) which is associated with ST258, ST307, and ST147 (Additional file 10: Fig S10C, Additional file 16: Table S4). The diversity of this collection of isolates is relatively low for ST, KL, and O-antigen, with Simpson’s diversity indices of 0.72, 0.85, and 0.53, respectively. In comparison with the NMH collection, NCBI isolates are highly clonal and likely the result of reporting bias of clonal outbreaks and highly antibiotic-resistant isolates. For example, 43 (68%) of ST307 bloodstream isolates were from a single hospital system (Houston-Methodist) [25]. Several temporal trends were apparent among the 963 isolates. Since 2008, ST258 isolates have comprised a substantial number of the sequences deposited in NCBI, but the proportional representation of this ST has dropped in recent years (Additional file 11: Fig S11). ST307 isolates were first submitted in 2011 and continued to be submitted through 2020 (Additional file 11: Fig S11). ST147 isolates were first submitted in 2015 but accounted for 35% of sequences uploaded in 2021 (Additional file 11: Fig S11). It is unclear whether these differences represent changes in the epidemiology of *K. pneumoniae* or simply reporting biases.

Among these publicly available United States bloodstream sequences, antimicrobial resistance was remarkably common; 91.7% of isolates contained an extended-spectrum beta-lactamase (ESBL) gene or a carbapenemase gene, compared to 17.3% of isolates in the NMH collection (Fig. 7; Additional file 16: Table S4). The large proportion of antimicrobial-resistant high-risk clones suggests that this collection suffers from sampling bias, with preferential submission of drug-resistant isolates potentially from clonal outbreaks.

We searched the genomes of the 963 isolates for acquired virulence genes. We identified genes required for the biosynthesis of yersiniabactin in 345 (35.8%) isolates, with the most common variants being *ybt17* (n = 179), *ybt10* (n = 42), and *ybt9* (n = 32) (Tables 1; Additional file 16: Table S4). Five isolates were predicted to contain a *ybt4-*containing plasmid (Additional file 12: Fig S12C). Colibactin biosynthetic genes were identified in 194 (20%) isolates, with *clb3* variants being the most common. NMH isolates had more diverse variants of *ybt* than those found in the NCBI collection, suggesting that the *ybt* variants in the NCBI collection were largely driven by ICE*Kp10*-containing ST258 isolates.

In addition to *ybt* and *clb*, the hvKP-associated loci *iuc, iro, rmpADC,* and *rmpA2* were also examined. *iuc* was identified in 32 (3.3%), *iro* in 9 (0.9%), *rmpADC* in 14 (1.5%), and *rmpA2* in 21 (2.2%) of the genomes (Table 1; Additional file 16: Table S4). The 32 *iuc*^+^ isolates were all predicted to contain a plasmid that fell into one of seven distinct groups, which in turn formed five plasmid network clusters (Additional file 12: Fig S12). Examination of the first of these clusters (Additional file 12: Fig. S12A) indicated that it was closely related to pK2044. A second cluster corresponded to KP52.145pII, a third to pTK421, and a fourth to aerobactin-encoding *Escherichia coli* plasmids (Additional file 12: Fig S12). A total of 25 (2.6%) isolates contained hypervirulence-associated lineages of *iuc* (*iuc1* or *iuc2*), and either *rmpA* or *rmpA2* indicating they are likely hvKP (Additional file 12: Fig S12A; Table 1; Additional file 16: Table S4). Among these, 18 isolates contained carbapenamase genes, indicating that these isolates are potential “convergent” isolates or carbapenem resistant-hvKP (Tables 1; Additional file 16: Table S4). Fourteen isolates were predicted to contain a hybrid antibiotic resistance-virulence plasmid (Additional file 12: Fig S12A) similar to those described previously in Europe [106, 107]. These data support the conjectures that hvKP strains are circulating in the United States and that some of these strains are both antibiotic-resistant and hypervirulent [67].

## Discussion

We examined consecutive *K. pneumoniae* bloodstream isolates at a single United States medical center and found that they had significant diversity in sequence type, capsule type, antimicrobial resistance genes, and virulence factors. In total, there were 75 distinct sequence types in our collection of 104 isolates, demonstrating that genetically diverse *K. pneumoniae* strains have the potential to cause invasive infections. As expected, the majority (96.2%) were cKP isolates, of which 16.3% were MDR. However, hvKP isolates comprised 3.8% of this collection. These findings indicate that most *K. pneumoniae* strains at NMH are antibiotic-susceptible but that a substantial number of MDR and hvKP strains are also present.

MDR cKP pose a considerable therapeutic challenge to clinicians in that the delay in administration of active antimicrobial therapy may lead to poor outcomes [108, 109]. The prevalence of MDR cKP bloodstream infections varies dramatically globally ranging from 18% to -61% [110–116]. Our finding that 16.3% of *K. pneumoniae* bloodstream isolates were MDR and 11.5% XDR underscores the difficulty in choosing empiric antibiotic therapy for patients at risk for *K. pneumoniae* bacteremia. Much of this resistance was driven by ESBL (11.5%) and carbapenemase (8.7%) genes found on mobile elements. These proportions agree with a report from the National Healthcare Safety Network, which found that 5–25% of *Klebsiella* species cultured from patients in the United States between 2015 and 2017 were resistant to carbapenems [117]. In our study, a substantial number of MDR isolates (70.6%) were high-risk clones, confirming the role of these widely disseminated lineages in driving antibiotic resistance [13, 118]. Recently, two large studies evaluated the molecular characteristics of either ESBL or carbapenem-resistant isolates from U.S. hospitalized patients and found that high-risk clones ST258 and ST307 accounted for 27–64% and 7–36% of these isolates, respectively [25, 119]. At NMH, ST258 was the most abundant ST and accounted for 6% of isolates, yet only two ST307 isolates were identified among bloodstream isolates. ST258 isolates are split into two distinct sub-lineages based on capsule type and KPC allele; KL107 is associated with KPC-3 while KL106 is associated with KPC-2 [120]. Both sub-lineages are found in high abundance in the NCBI collection (Fig. 7). At NMH, ST258 isolates are primarily part of the KL107, KPC-3 sub-lineage (Additional file 13: Table S1, Fig. 7). These observations illustrate the burden of antibiotic resistance and clonal outbreaks of high-risk clones among U.S. *K. pneumoniae* isolates.

Intriguingly, four (3.8%) of the isolates in the NMH collection had features of hvKP. These isolates belonged to the established hvKP lineages ST23 and ST380 and to the re-emerging lineage ST66. Each harbored a plasmid similar to one of the two previously described hvKp virulence plasmids (pK2044 or KP52.145pII) [41–43]. These plasmids contained multiple hvKP-associated genetic loci (*iuc, iro,* and *rmpA*). The isolates were hmv and had LD_50_ values in mice consistent with hvKp (Fig. 6; Table 2). Three of the isolates (KPN49, KPN115, and KPN165) caused severe community-acquired infections with abscess formation (2 hepatic and 1 lung). Taken together, these data indicate that these four isolates are indeed hvKP and that multiple strains of hvKp are circulating in the Chicago region.

One strength of our approach is the use of a mouse model to quantify the in vivo virulence of potential hvKP isolates. Virulence in mice accurately distinguishes hvKP from cKP strains [40]. This approach allowed us to show that KPN23, a non-hmv isolate containing *iuc* but not *rmpA, rmpA2,* or *iro*, had an intermediate level of virulence between hvKP and cKP (Fig. 6). These findings suggest that some *K. pneumoniae* strains may not fall neatly into the categories of cKP or hvKP but may have more nuanced genotypes and phenotypes. Our experiments also demonstrated that KPN80, an hmv isolate lacking *iuc, rmpA, rmpA2,* and *iro*, had a low level of virulence consistent with cKP strains (Fig. 6). The characteristics of this isolate support previous reports indicating that hmv alone does not accurately predict hvKP [64, 81]. Together, these observations suggest that inferences about hvKP should be made with caution when examining only a single bacterial gene or trait.

Our findings also have implications for the pathogenesis of hvKP. They suggest that hmv caused by *rmpA* (e.g., KPN49) has dramatically different pathogenic consequences than hmv caused by a capsule locus mutation (KPN80) (Fig. 6; LD_50_ of 10^1.5^ vs. 10^8.1^ CFU, respectively). Likewise, our finding that increased levels of siderophore secretion were associated with increased in vivo virulence (Additional file 9: Fig S9) suggests that siderophores also contribute to the overall hvKP virulence phenotype. We note that these results are only associations, and that experiments using genetically defined mutants will be necessary to unravel the relative contributions of these genetically linked hvKP genes to virulence.

Knowledge of the epidemiology of hvKP has been hampered by the lack of a consensus definition for these strains. The two previously published U.S. surveillance studies dealt with this difficulty in different ways. In 2010, a study of 64 *K. pneumoniae* bloodstream isolates from two hospitals in the greater Houston area screened for isolates containing *rmpA* or *wzy_K1* (a biomarker for a K1 capsule type) [39]. They identified 4 (6.3%) potential hvKP isolates using this approach. In 2018, a larger study in New York City examined *K. pneumoniae* isolates from 462 patients for the presence of both *rmpA* and *iucA* [37]. This study identified 17 (3.7%) potential hvKP isolates containing these two genes. In contrast, a 2013 report from China, where hvKP is endemic, described an hvKP prevalence of 37.8% using the presence of the *iuc* locus to define hvKP [65]. Similarly, a recent genomic surveillance study of seven countries in South and Southeast Asia analyzed the sequences of 365 *K. pneumoniae* bloodstream isolates collected from 2010–2017 [69]. Sequencing revealed that 95 (26%) were *iuc*^+^ . Of these, 63 (17%) also contained either *rmpA* or *rmpA2*. Precise definitions that accurately distinguish hvKP from cKP and at the same time capture the diversity of hvKP will be necessary in future studies to evaluate the prevalence and clinical consequences of hvKP infections.

Thus far, antimicrobial-resistant hvKp infections appear to be rare in the United States, as only 1.9% of bloodstream isolates from NCBI had features of carbapenem-resistant hvKP and none of the *iuc*^+^ isolates identified at NMH contained accessory antimicrobial resistance genes. However, the KP52.145pII-like plasmids of KPN49 and KPN165 have acquired putative conjugation genes, suggesting that these hypervirulence plasmids are capable of horizontal transmission. Indeed, KPN49 and KPN165 are phylogenetically distinct (ST66 and ST380, Additional file 8: Fig S8), consistent with horizontal transfer of this plasmid. This finding is worrisome and suggests that these plasmids may be prone to transfer to other *K. pneumoniae* strains, including MDR strains. Additional studies are necessary to demonstrate whether these conjugation genes are functional and indeed confer transmissibility on this hypervirulence plasmid.

## Conclusions

In this study, consecutive *K. pneumoniae* bloodstream isolates were collected from 2015-2017 at a single U.S. medical center. Whole genome sequencing revealed that these isolates are diverse in sequence type, capsule type, and virulence gene content. The majority (79.9%) of isolates were cKP strains susceptible to most antibiotics. However, MDR cKP isolates and hvKP isolates comprised 16.3% and 3.8% of this collection, respectively. These findings confirm previous reports that MDR cKP are common in the U.S. and that several hvKP strains are also circulating in the Chicago region. Examination of the larger NCBI database confirmed that strains likely to be hvKP are circulating in the U.S.

## Supplementary Information

**Additional file 1:** **Figure S1. **A variety of *Klebsiella spp*. were identified among NMH bloodstream isolates. Maximum likelihood phylogenetic tree generated from core genome SNP loci in 140 *Klebsiella spp*. bloodstream isolates. The tree has a truncated outlier branch for *Klebsiella oxytoca* (yellow). The scale bar represents genetic distance (number of substitutions per site).

**Additional file 2:** **Figure S2**. NMH *K. pneumoniae* bloodstream isolates are highly diverse in ST, KL, and O-antigen. Numbers of genomes with each corresponding ST **(A)**, KL **(B)**, or O-antigen type **(C)** were determined using *Kleborate* and *Kaptive*.

**Additional file 3:** **Figure S3. **Antimicrobial resistance phenotypes of *K. pneumoniae* bloodstream isolates from NMH. The percentage of isolates resistant (solid bars) or intermediately susceptible (cross-hatched bars) to the indicated antibiotics are shown. Antibiotic classes are grouped by color.

**Additional file 4:** **Figure S4**. Beta-lactam-resistant isolates contain a variety of extended-spectrum beta-lactamase or carbapenemase genes. Numbers of genomes containing the indicated beta-lactamase genes present in isolates resistant to ceftriaxone **(A)**, meropenem **(B)**, ampicillin/sulbactam **(C)**, aztreonam **(D)**, cefazolin **(E)**, or cefepime **(F)** are plotted. “- “ indicates isolates without an extended-spectrum beta-lactamase gene.

**Additional file 5:** **Figure S5. **Predicted plasmids containing virulence genes among NMH bloodstream isolates. Putative plasmids predicted to contain *iuc* or *ybt* loci were inferred and clustered using MOB-suite. Individual nodes represent a plasmid, and different colors represent a different plasmid group. Two plasmids are connected by an edge if their Jaccard index is ≥ 0.95. Networks were graphed using Cytoscape.

**Additional file 6:** **Figure S6. **pKPN49_2, pKPN165_1, pTK421_2, and pKPN23_1 have little similarity to pK2044. Plasmid sequences were aligned to pK2044 using blast ring image generator (BRIG). A sequence identity threshold of 85% was used. Aerobactin biosynthesis genes are indicated with green arrows, salmochelin with orange arrows, *rmpA* with a blue arrow, and *rmpA2* with a red arrow.

**Additional file 7: Figure S7. **The bloodstream isolate KPN23 harbors a plasmid containing aerobactin biosynthesis genes. The indicated plasmids were aligned to a plasmid identified in strain KPCTRSRTH01_p2 **(A)** or to pKPN23_1 **(B)** using BRIG. A sequence identity threshold of 85% was used. Aerobactin biosynthesis genes are indicated with green arrows. Plasmid accession numbers for previously published plasmids are listed in Table S7.

**Additional file 8:** **Figure S8. **Core genome phylogenetic tree of hypervirulent *K. pneumoniae* isolates. A maximum likelihood phylogenetic tree was generated from core genome SNP loci in hvKP isolates from United States hospitals and selected global reference isolates. Isolates labeled in red text are NMH bloodstream isolates from this study. Clonal group 23 sublineage 1 (CG23-1) isolates are indicated in a red box. Sequence types (ST) and capsule types (KL) are indicated. The presence of virulence genes is indicated next to each isolate: *ybt* = yersiniabactin biosynthesis loci, *clb* = colibactin biosynthesis loci, *rmpADC* = mucoid regulator operon, *rmpA2* = regulator of mucoid phenotype 2, *iuc* = aerobactin biosynthesis genes, *iro* = salmochelin biosynthesis genes. KPPR1, KP52.145, SB5881, K180005, NCTC9494, NTUH-K2044, RJF999, and ED2 are isolates used as references (accession numbers are listed in Table S7).

**Additional file 9**: **Figure S9. **Siderophore activity and virulence of NMH bloodstream isolates. Siderophore activity and virulence is plotted for each isolate (**A**) and against each other (**B**): left y-axis **(A,** purple triangles**)** or x-axis **(B)**. In (**B**), aerobactin-positive (*iuc*^+^) strains are labeled in purple **(B)**. Virulence was measured by LD_50_ values in a mouse model of pneumonia. Siderophore activity was measured by dipyridyl equivalents (mM) detected in cell free supernatants of bacterial cultures. Number of mice and doses used to determine LD_50_ values are listed in Table S8.

**Additional file 10: Figure S10.** Sequence, capsule, and O-antigen types of NCBI *K. pneumoniae* bloodstream isolates. Numbers of genomes with each corresponding ST **(A)**, KL **(B)**, or O-antigen type **(C)** were determined using *Kleborate* and *Kaptive*. 1LV, 2LV, and 3LV indicate 1, 2, or 3 SNPs from a previously published ST.

**Additional file 11: Figure S11.** Proportion of bloodstream genomes deposited to NCBI that were high-risk clones. Percent of genomes with the indicated sequence types are graphed for each year from 2007 – 2021. Numbers of total sequences deposited each year are listed directly above the graph. Sequence types were determined using Kleborate.

**Additional file 12: Figure S12. **Predicted virulence plasmids carried by NCBI bloodstream isolates. Putative plasmids predicted to contain *iuc* or *ybt* genes were inferred and clustered using MOB-suite: *iuc1* **(A)**, *iuc5* and *iuc* unknown **(B)**, *ybt4* **(C)**, and *iuc2* **(D)**. Individual nodes represent a plasmid, and different colors represent a different plasmid group. Two plasmids are connected by an edge if their Jaccard index is ≥ 0.95. Networks were graphed using Cytoscape. Hybrid ESBL-virulence or carbapenemase-virulence plasmids are outlined with a dashed line and labeled with the type of beta-lactamase. Networks were graphed using Cytoscape.

**Additional file 13**: **Table S1**. Detailed characteristics of *Klebsiella pneumoniae* isolates.

**Additional file 14**: **Table S2**. Features of in silico-predicted plasmids.

**Additional file 15**: **Table S3**. Characteristics of plasmids predicted to contain ESBL or carbapenemase genes.

**Additional file 16**: **Table S4**. Characteristics of NCBI bloodstream isolates.

**Additional file 17**: **Table S5**. Sequenced isolates from this study.

**Additional file 18**: **Table S6**. Complete genomes from this study.

**Additional file 19**: **Table S7**. Reference genomes.

**Additional file 20**: **Table S8**. Number of mice dosing and mortality.

## Data Availability

The whole-genome assemblies have been deposited at GenBank under BioProject number PRJNA788509. Assembly accession numbers are included in Additional file 16: Table S4, Additional file 17: Table S5, and Additional file 18: Table S6. *Kleborate* and MIC data are included in Additional file 13: Table S1.

## Abbreviations

ST: Sequence type
KL: Capsule loci
MDR: Multidrug-resistance
XDR: Extensively drug-resistant
ESBL: Extended-spectrum beta-lactamase
cKP: Classical *Klebsiella pneumoniae*
hvKP: Hypervirulent *Klebsiella pneumoniae*
NMH: Northwestern Memorial Hospital
PAL-1: Pathogenicity loci 1
PAL-2: Pathogenicity loci 2
ICE: Integrative and conjugative element
hmv: Hypermucoviscous
KpSC: *Klebsiella pneumoniae* species complex
MIC: Minimum inhibitory concentration
TMP/SMX: Trimethoprim and sulfamethoxazole
AME: Aminoglycoside modifying enzyme
LD50: 50% Lethal dose
CAS: Chrome-azurol S
NCBI: National Center for Biotechnology Information
