# Supplementary figures and images for: Genomic surveillance for multidrug-resistant or hypervirulent Klebsiella pneumoniae among United States bloodstream isolates
Source: BMC Infect Dis. 2022 Jul 7;22:603. doi: 10.1186/s12879-022-07558-1 (PMC9263067; doi:10.1186/s12879-022-07558-1)

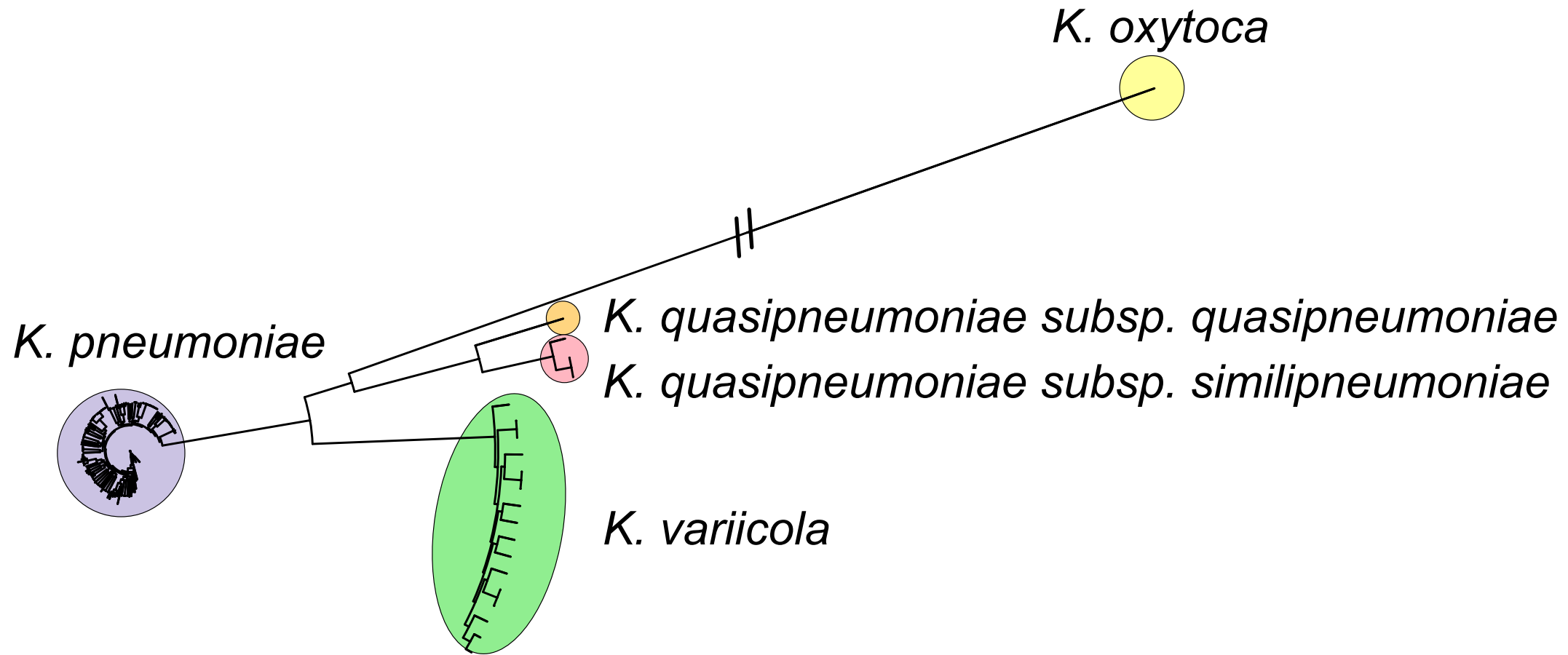

Supplement: Supplementary file 1 — Additional file 1: Figure S1. A variety of Klebsiella spp. were identified among NMH bloodstream isolates. Maximum likelihood phylogenetic tree generated from core genome SNP loci in 140 Klebsiella spp. bloodstream isolates. The tree has a truncated outlier branch for Klebsiella oxytoca (yellow). The scale bar represents genetic distance (number of substitutions per site). [file 12879_2022_7558_MOESM1_ESM.pdf]

A

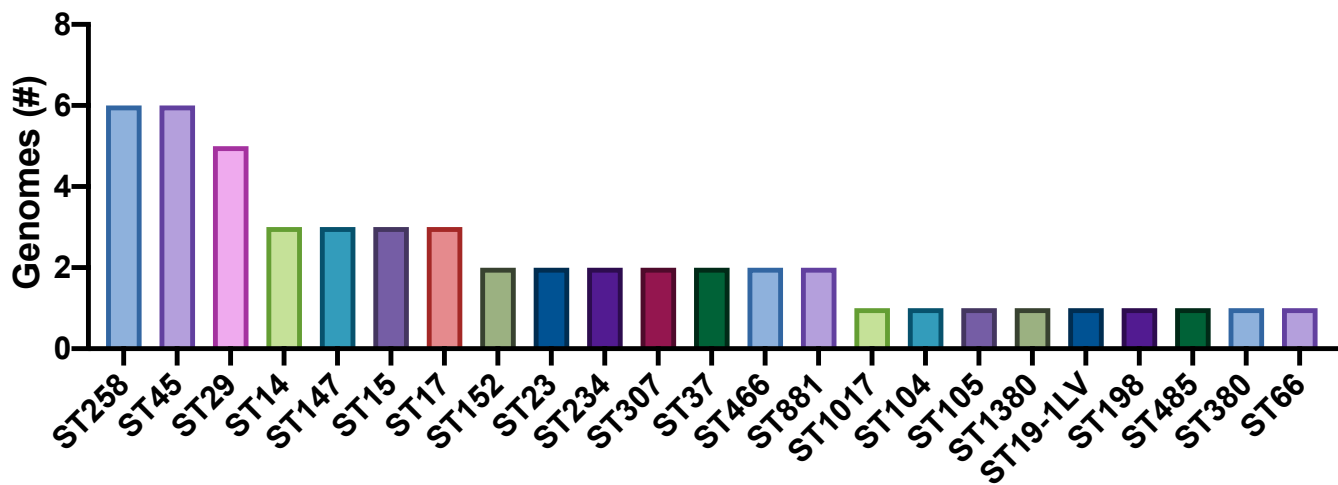

B

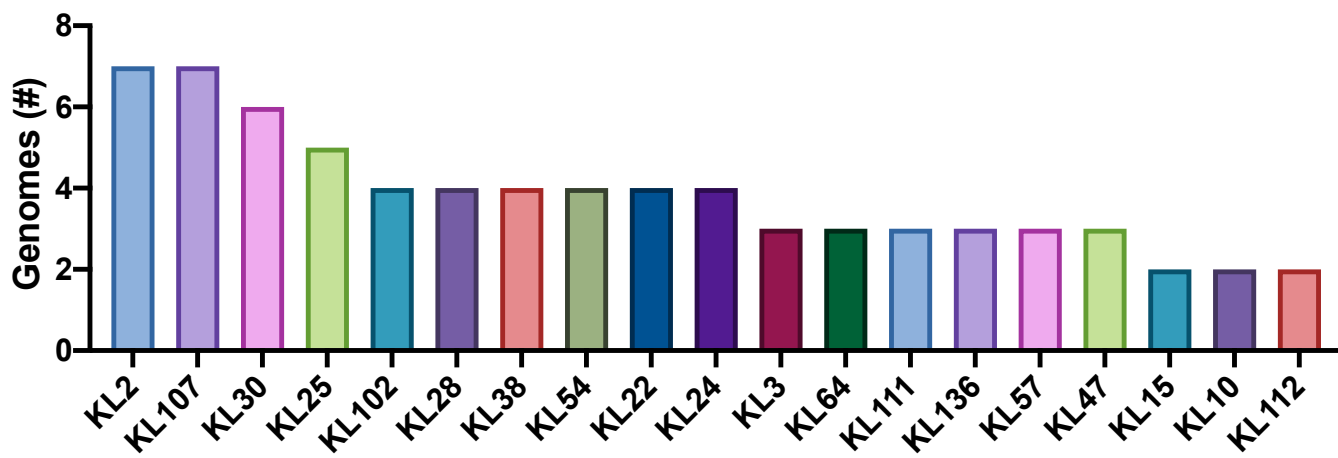

C

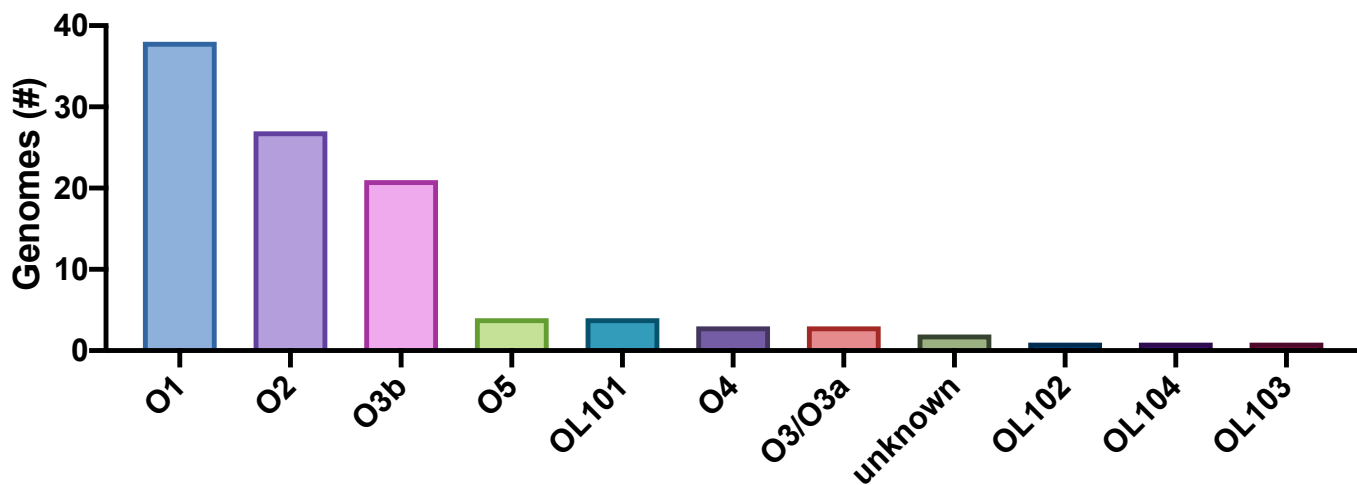

Supplement: Supplementary file 2 — Additional file 2: Figure S2. NMH K. pneumoniae bloodstream isolates are highly diverse in ST, KL, and O-antigen. Numbers of genomes with each corresponding ST (A), KL (B), or O-antigen type (C) were determined using Kleborate and Kaptive. [file 12879_2022_7558_MOESM2_ESM.pdf]

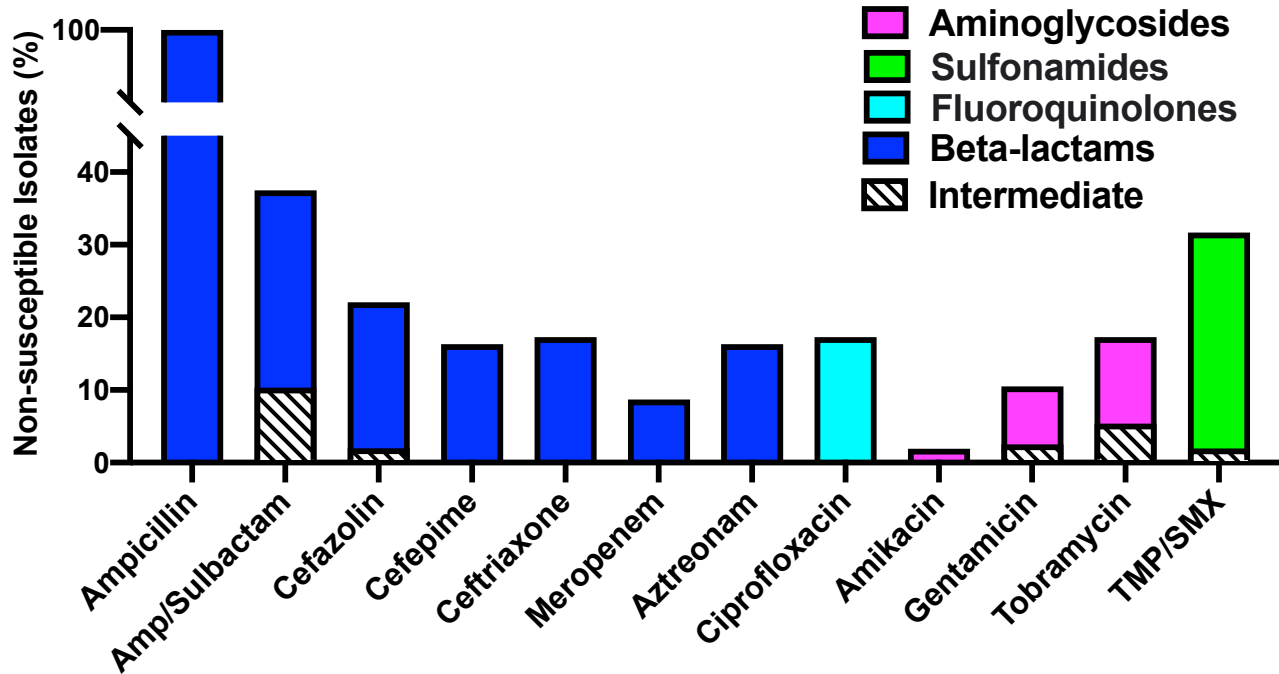

Supplement: Supplementary file 3 — Additional file 3: Figure S3. Antimicrobial resistance phenotypes of K. pneumoniae bloodstream isolates from NMH. The percentage of isolates resistant (solid bars) or intermediately susceptible (cross-hatched bars) to the indicated antibiotics are shown. Antibiotic classes are grouped by color. [file 12879_2022_7558_MOESM3_ESM.pdf]

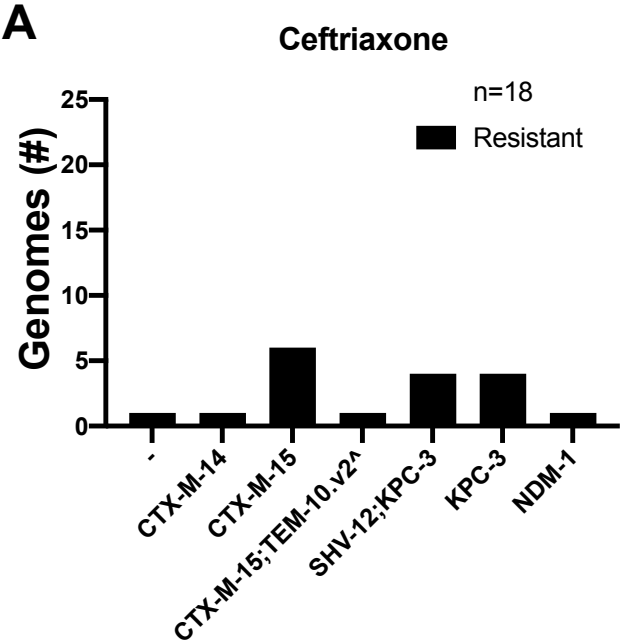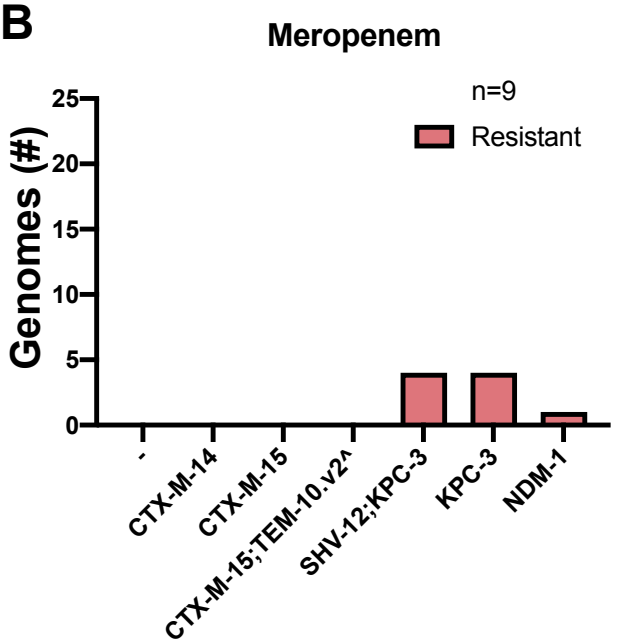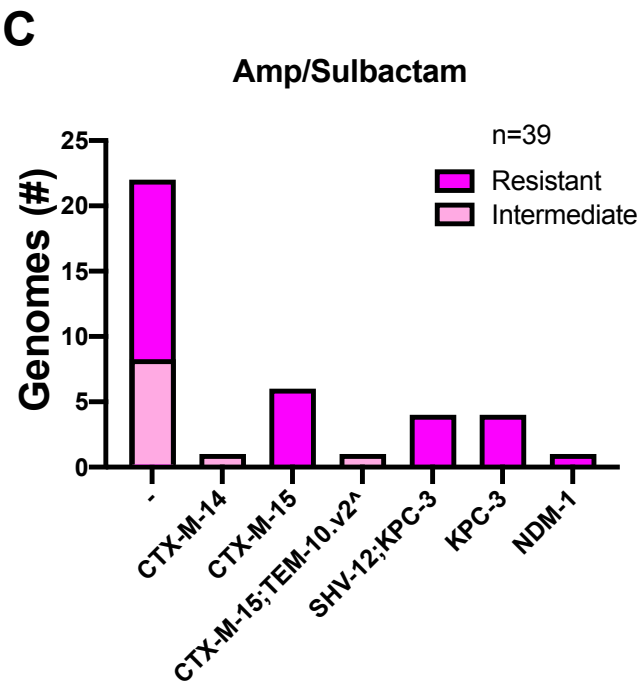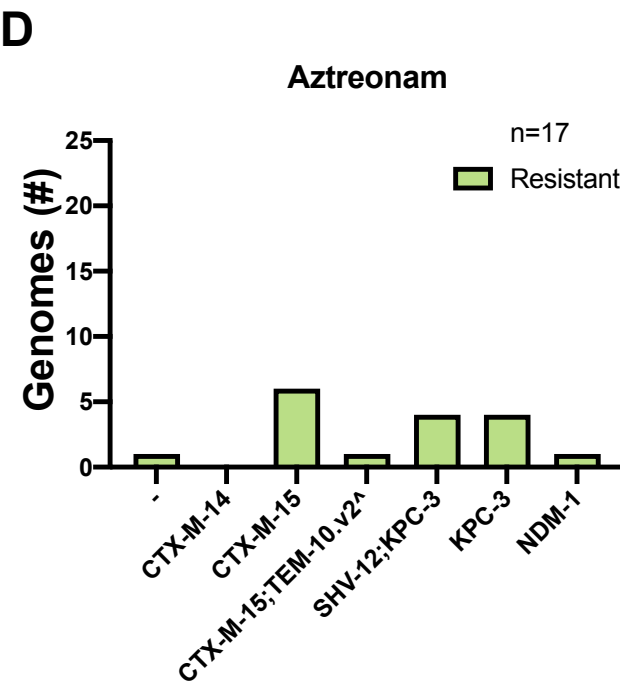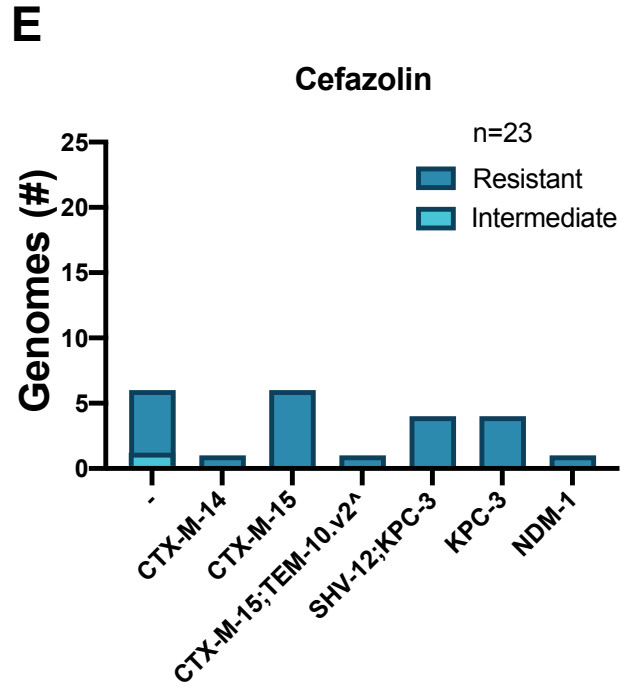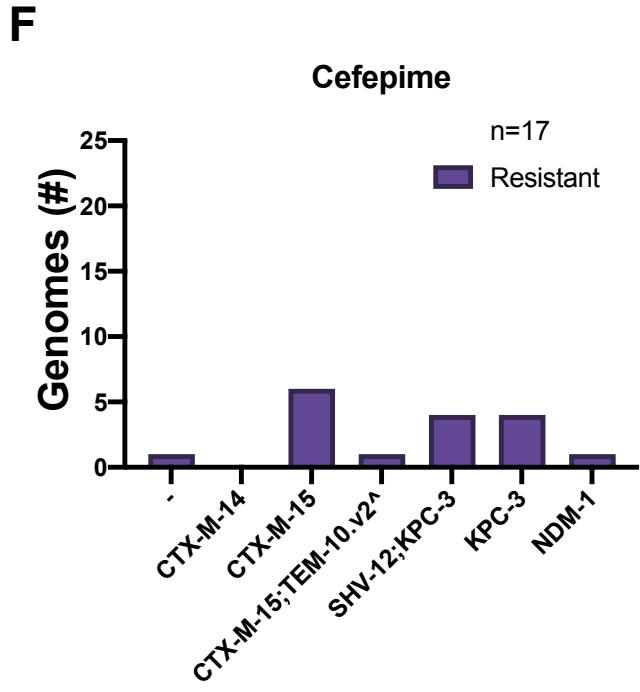

Supplement: Supplementary file 4 — Additional file 4: Figure S4. Beta-lactam-resistant isolates contain a variety of extended-spectrum beta-lactamase or carbapenemase genes. Numbers of genomes containing the indicated beta-lactamase genes present in isolates resistant to ceftriaxone (A), meropenem (B), ampicillin/sulbactam (C), aztreonam (D), cefazolin (E), or cefepime (F) are plotted. “- “ indicates isolates without an extended-spectrum beta-lactamase gene. [file 12879_2022_7558_MOESM4_ESM.pdf]

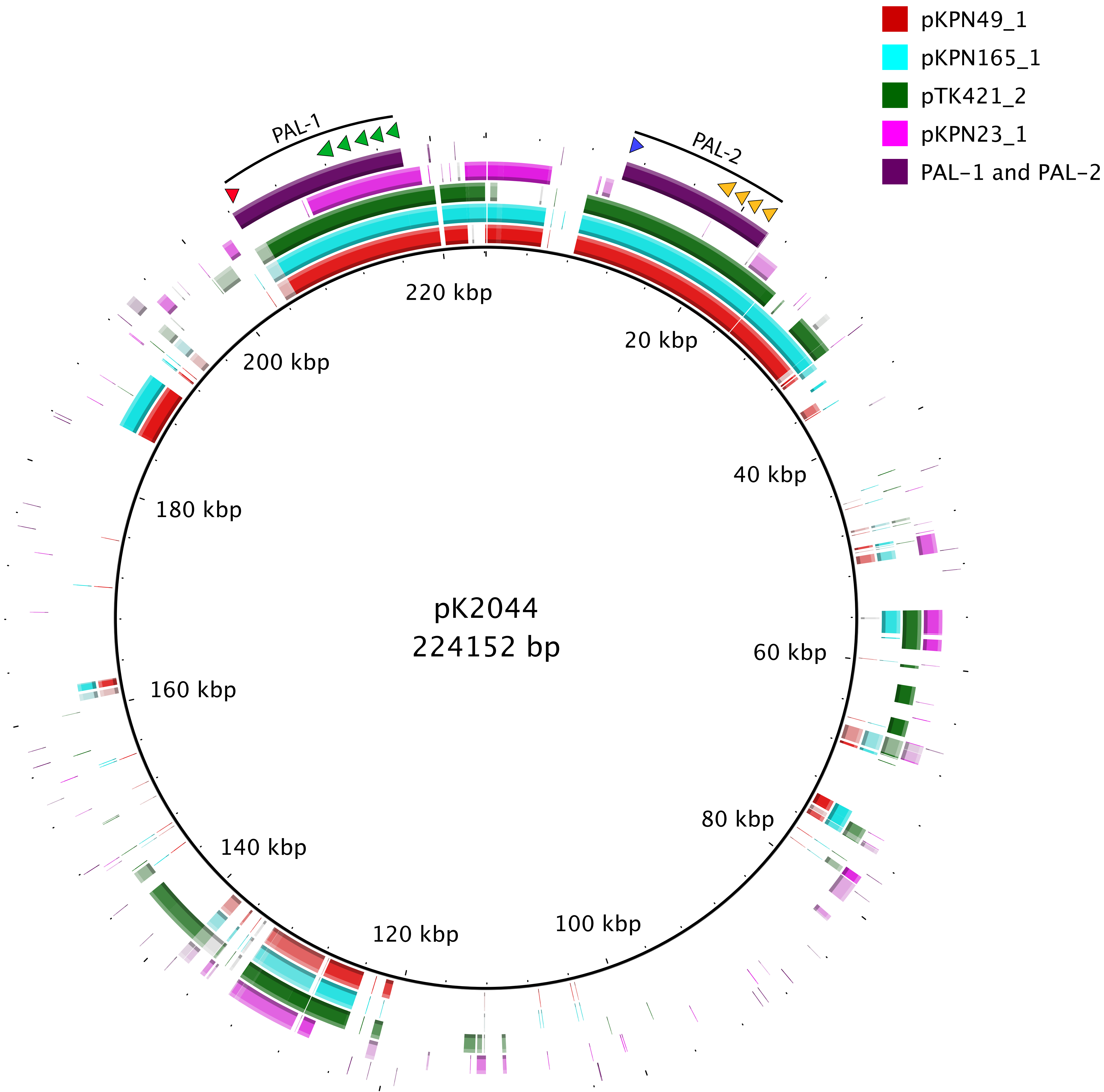

Supplement: Supplementary file 6 — Additional file 6: Figure S6. pKPN49_2, pKPN165_1, pTK421_2, and pKPN23_1 have little similarity to pK2044. Plasmid sequences were aligned to pK2044 using blast ring image generator (BRIG). A sequence identity threshold of 85% was used. Aerobactin biosynthesis genes are indicated with green arrows, salmochelin with orange arrows, rmpA with a blue arrow, and rmpA2 with a red arrow. [file 12879_2022_7558_MOESM6_ESM.pdf]

A

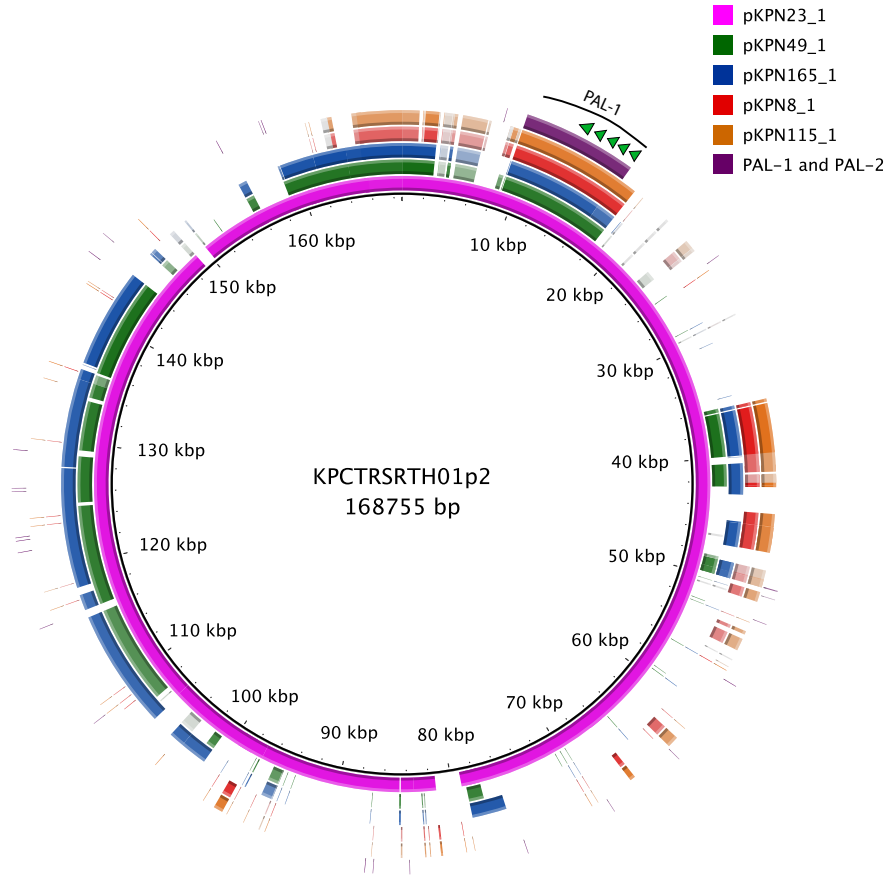

B

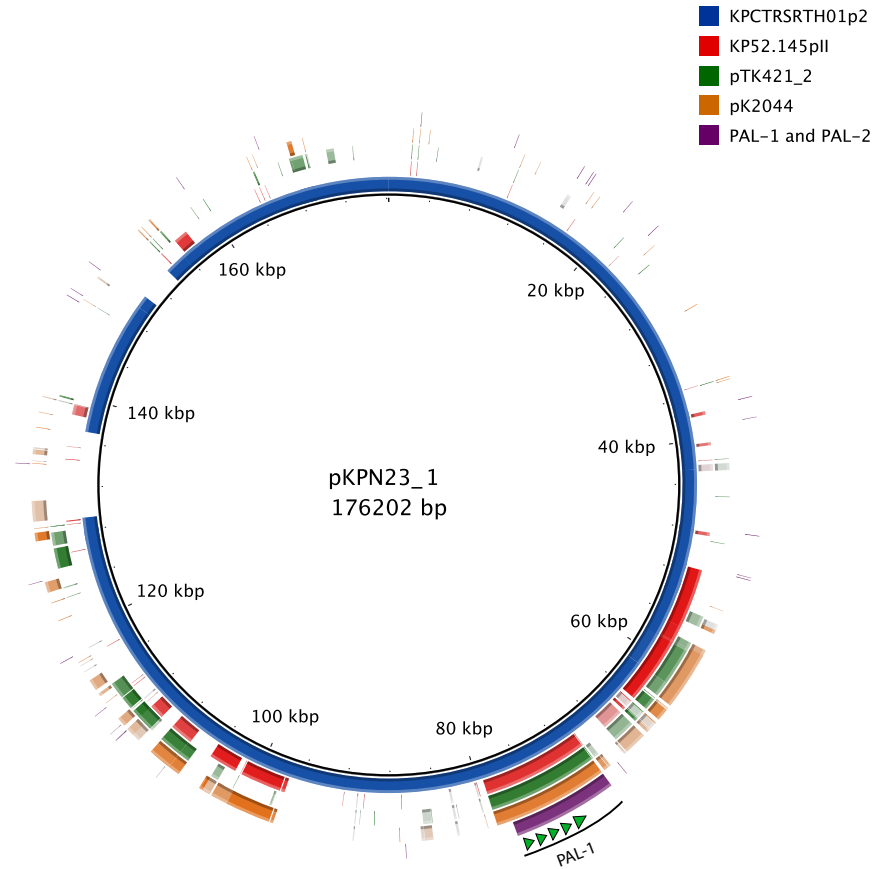

Supplement: Supplementary file 7 — Additional file 7: Figure S7. The bloodstream isolate KPN23 harbors a plasmid containing aerobactin biosynthesis genes. The indicated plasmids were aligned to a plasmid identified in strain KPCTRSRTH01_p2 (A) or to pKPN23_1 (B) using BRIG. A sequence identity threshold of 85% was used. Aerobactin biosynthesis genes are indicated with green arrows. Plasmid accession numbers for previously published plasmids are listed in Table S7. [file 12879_2022_7558_MOESM7_ESM.pdf]

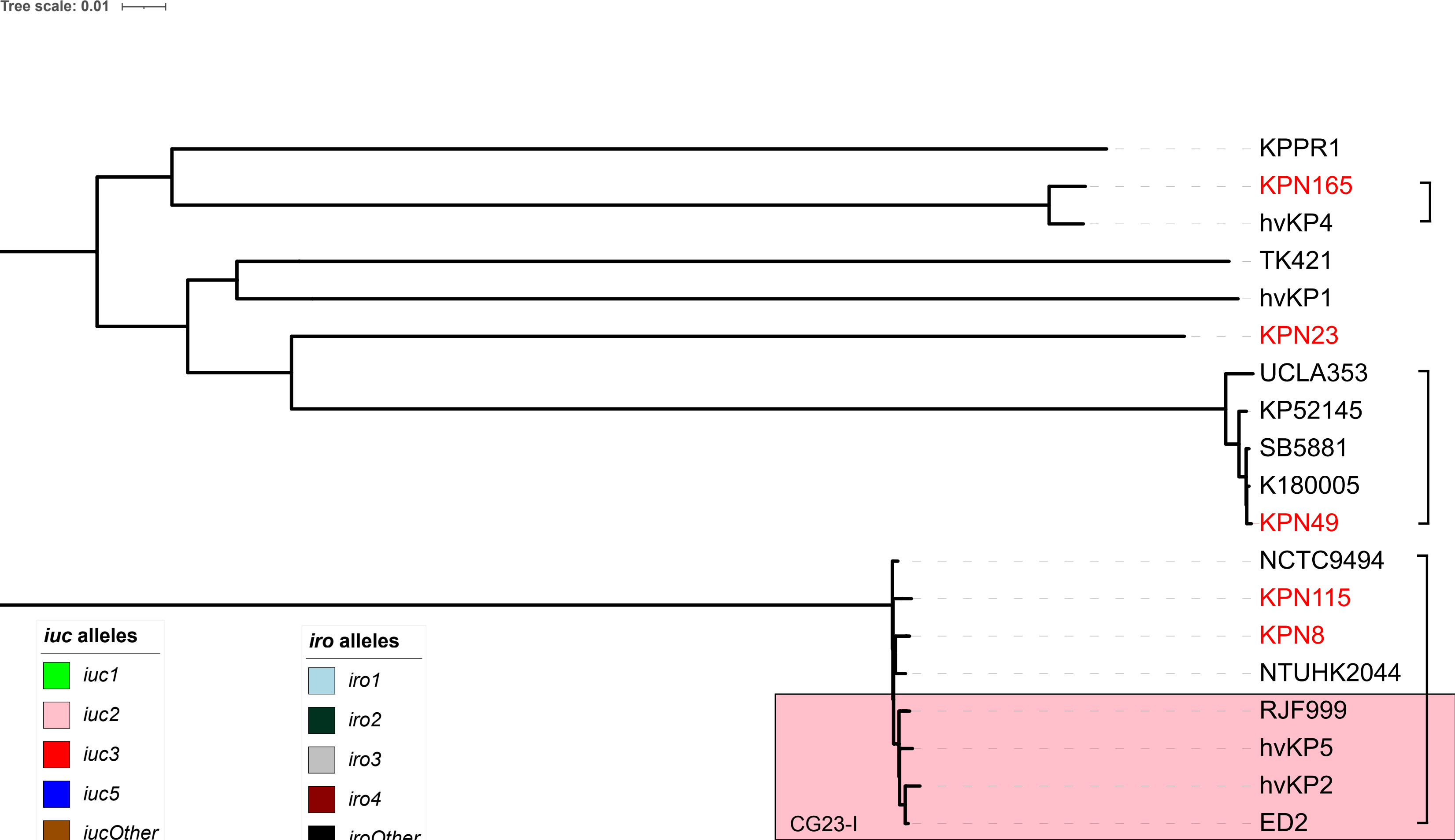

| ST    | KL   | ybt | clb | rmpADC | rmpA2 | iro | iuc |
|-------|------|-----|-----|--------|-------|-----|-----|
| ST493 | KL2  |     |     |        |       |     |     |
| ST380 | KL2  |     |     |        |       |     |     |
| ST34  | KL20 |     |     |        |       |     |     |
| ST86  | KL2  |     |     |        |       |     |     |
| ST881 | KL2  |     |     |        |       |     |     |
| ST66  | KL2  |     |     |        |       |     |     |
| ST23  | KL1  |     |     |        |       |     |     |

Supplement: Supplementary file 8 — Additional file 8: Figure S8. Core genome phylogenetic tree of hypervirulent K. pneumoniae isolates. A maximum likelihood phylogenetic tree was generated from core genome SNP loci in hvKP isolates from United States hospitals and selected global reference isolates. Isolates labeled in red text are NMH bloodstream isolates from this study. Clonal group 23 sublineage 1 (CG23-1) isolates are indicated in a red box. Sequence types (ST) and capsule types (KL) are indicated. The presence of virulence genes is indicated next to each isolate: ybt = yersiniabactin biosynthesis loci, clb = colibactin biosynthesis loci, rmpADC = mucoid regulator operon, rmpA2 = regulator of mucoid phenotype 2, iuc = aerobactin biosynthesis genes, iro = salmochelin biosynthesis genes. KPPR1, KP52.145, SB5881, K180005, NCTC9494, NTUH-K2044, RJF999, and ED2 are isolates used as references (accession numbers are listed in Table S7). [file 12879_2022_7558_MOESM8_ESM.pdf]

A

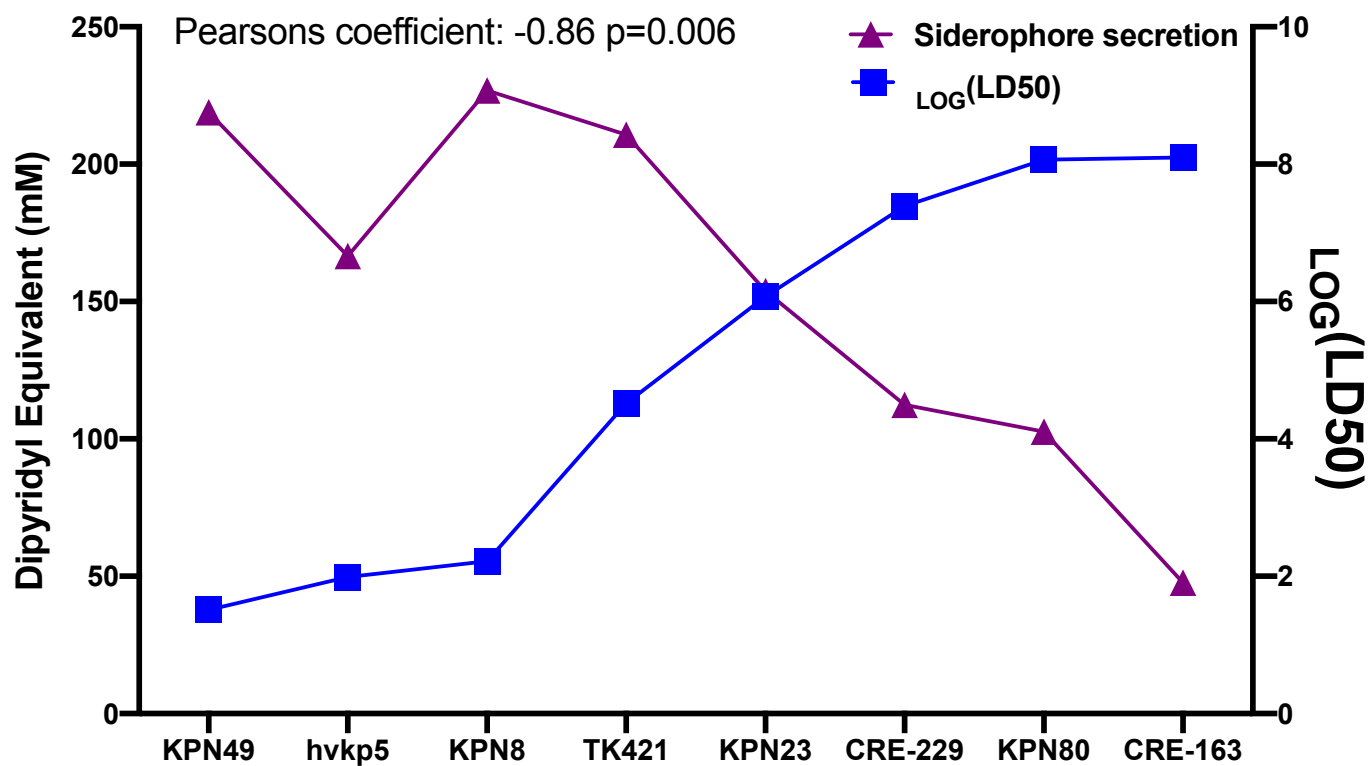

B

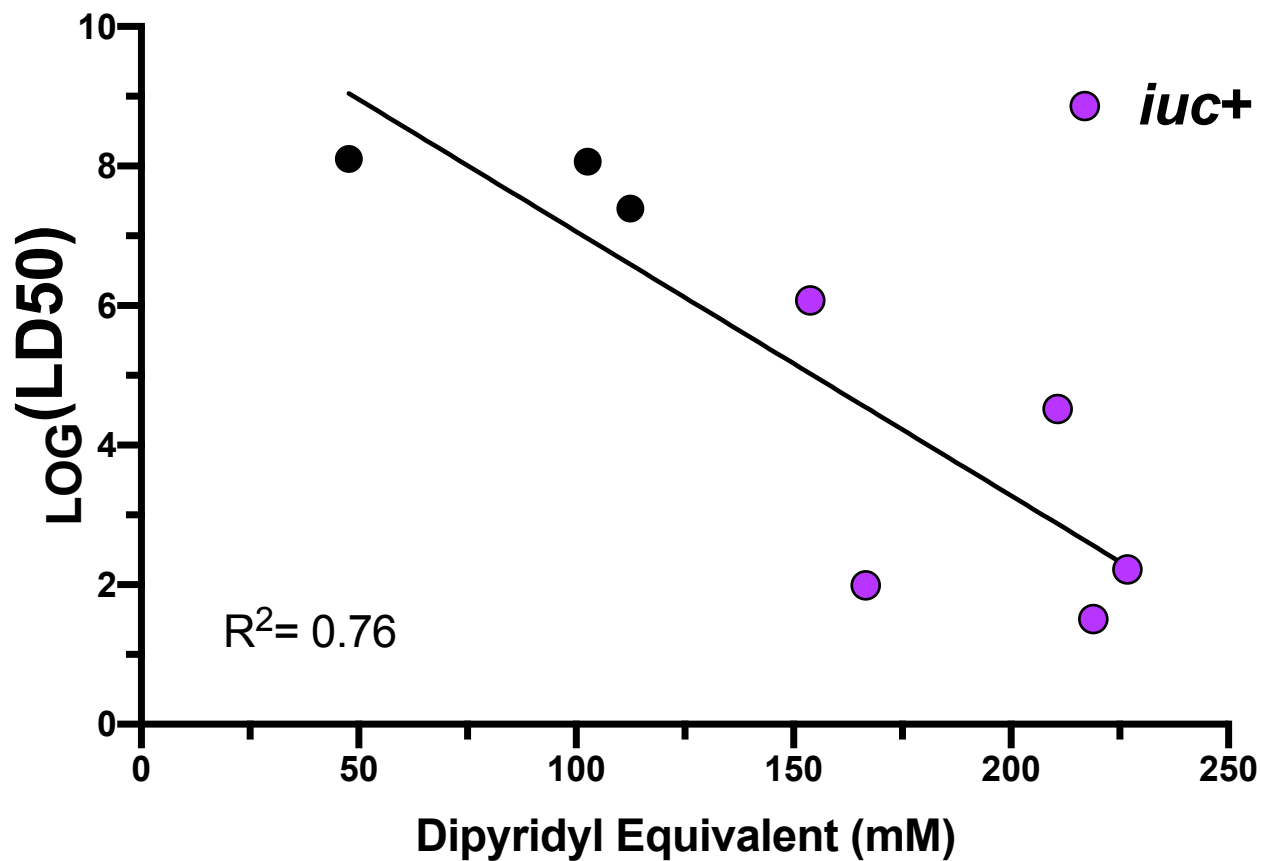

Supplement: Supplementary file 9 — Additional file 9: Figure S9. Siderophore activity and virulence of NMH bloodstream isolates. Siderophore activity and virulence is plotted for each isolate (A) and against each other (B): left y-axis (A, purple triangles) or x-axis (B). In (B), aerobactin-positive (iuc+) strains are labeled in purple (B). Virulence was measured by LD50 values in a mouse model of pneumonia. Siderophore activity was measured by dipyridyl equivalents (mM) detected in cell free supernatants of bacterial cultures. Number of mice and doses used to determine LD50 values are listed in Table S8. [file 12879_2022_7558_MOESM9_ESM.pdf]

A

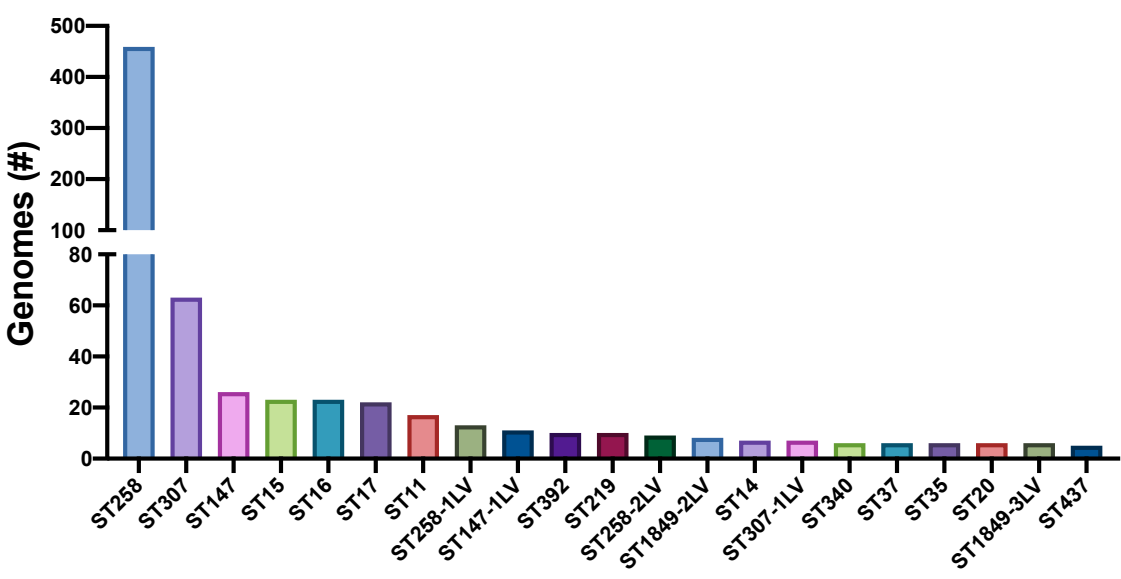

B

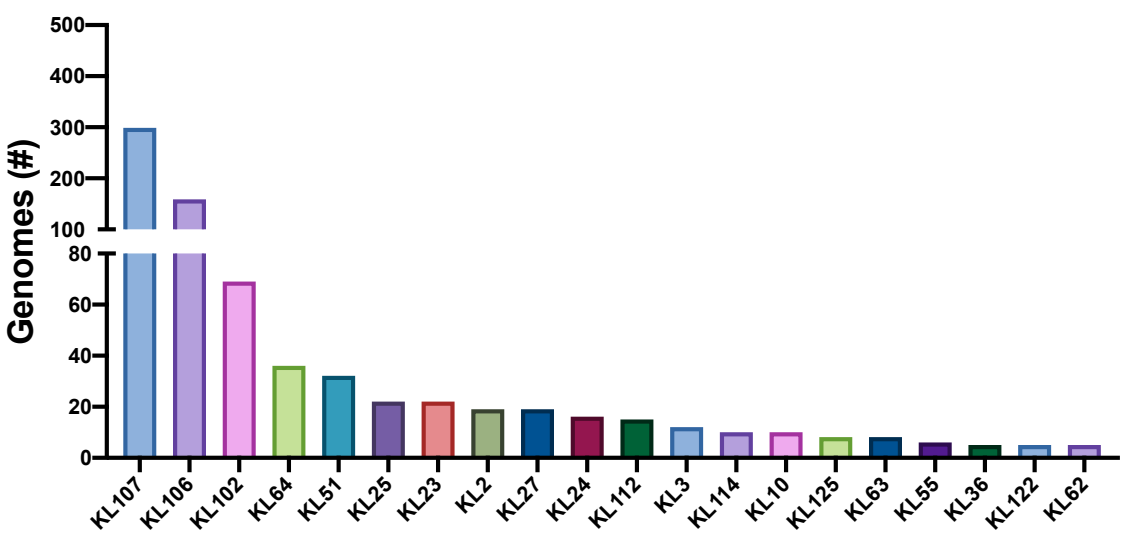

C

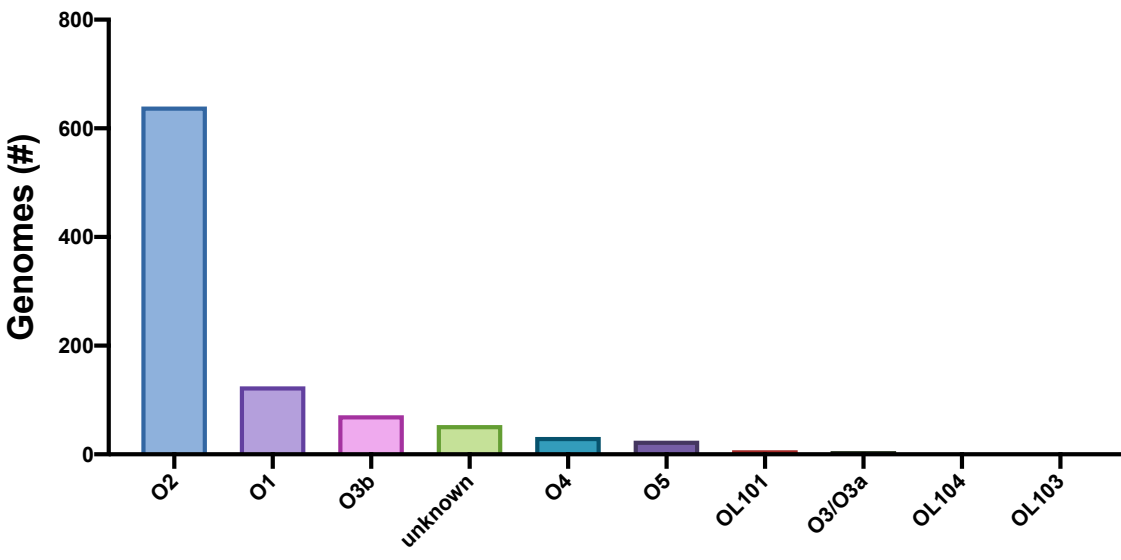

Supplement: Supplementary file 10 — Additional file 10: Figure S10. Sequence, capsule, and O-antigen types of NCBI K. pneumoniae bloodstream isolates. Numbers of genomes with each corresponding ST (A), KL (B), or O-antigen type (C) were determined using Kleborate and Kaptive. 1LV, 2LV, and 3LV indicate 1, 2, or 3 SNPs from a previously published ST. [file 12879_2022_7558_MOESM10_ESM.pdf]

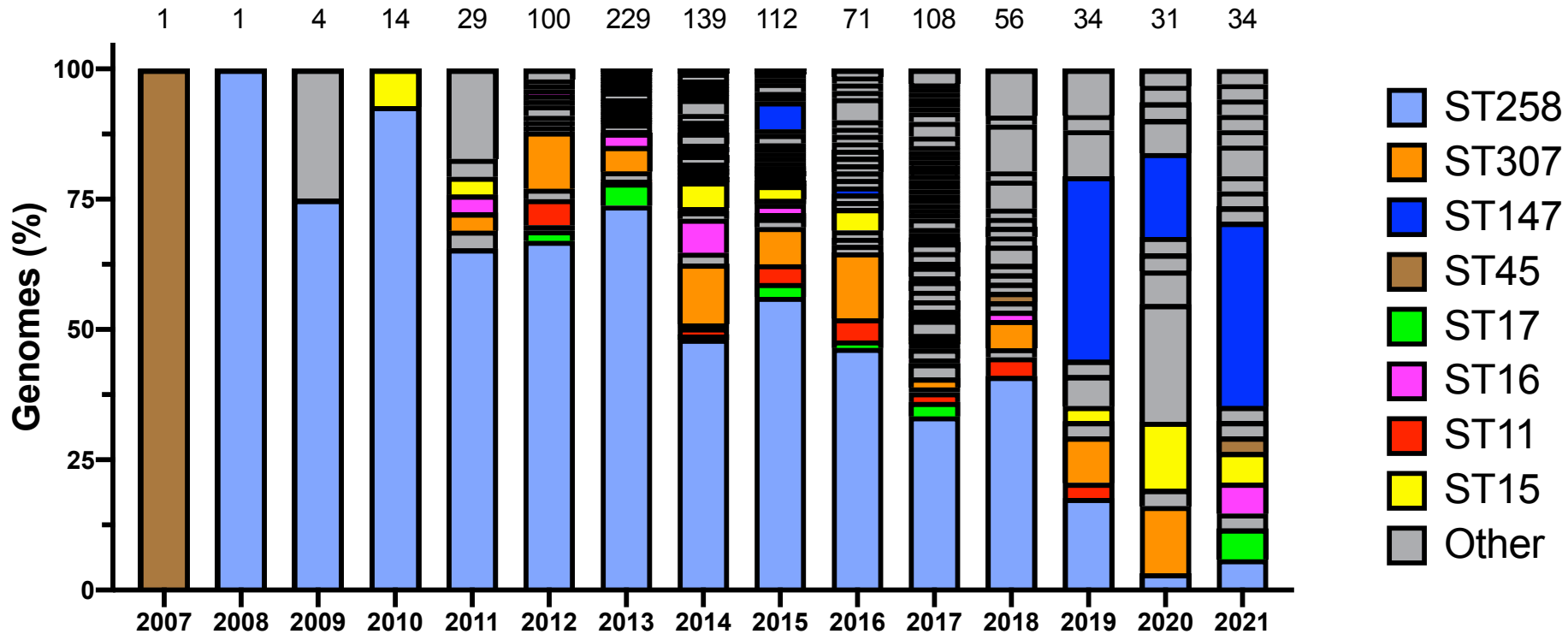

Supplement: Supplementary file 11 — Additional file 11: Figure S11. Proportion of bloodstream genomes deposited to NCBI that were high-risk clones. Percent of genomes with the indicated sequence types are graphed for each year from 2007 – 2021. Numbers of total sequences deposited each year are listed directly above the graph. Sequence types were determined using Kleborate. [file 12879_2022_7558_MOESM11_ESM.pdf]
