# Supplementary material for: Genomic surveillance for multidrug-resistant or hypervirulent Klebsiella pneumoniae among United States bloodstream isolates
Source: BMC Infect Dis. 2022 Jul 7;22:603. doi: 10.1186/s12879-022-07558-1 (PMC9263067; doi:10.1186/s12879-022-07558-1)
Supplement: Supplementary file 5 — Additional file 5: Figure S5. Predicted plasmids containing virulence genes among NMH bloodstream isolates. Putative plasmids predicted to contain iuc or ybt loci were inferred and clustered using MOB-suite. Individual nodes represent a plasmid, and different colors represent a different plasmid group. Two plasmids are connected by an edge if their Jaccard index is ≥ 0.95. Networks were graphed using Cytoscape. [file 12879_2022_7558_MOESM5_ESM.pdf]

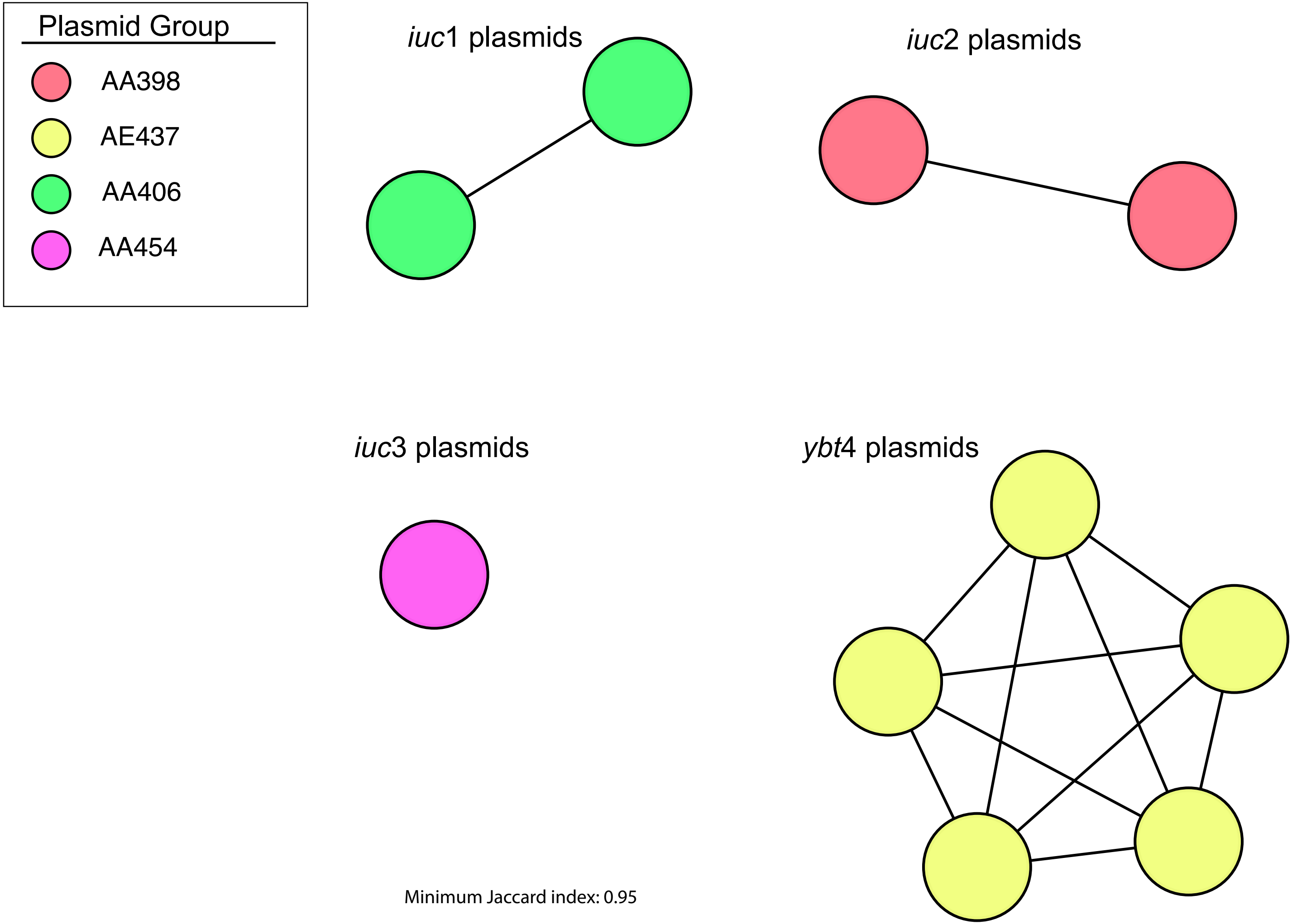
Plasmid Group
AA398
AE437
AA406
AA454
*iuc*1 plasmids
*iuc*2 plasmids
*iuc*3 plasmids
*ybt*4 plasmids
Minimum Jaccard index: 0.95
