# Supplementary material for: Genomic surveillance for multidrug-resistant or hypervirulent Klebsiella pneumoniae among United States bloodstream isolates
Source: BMC Infect Dis. 2022 Jul 7;22:603. doi: 10.1186/s12879-022-07558-1 (PMC9263067; doi:10.1186/s12879-022-07558-1)
Supplement: Supplementary file 12 — Additional file 12: Figure S12. Predicted virulence plasmids carried by NCBI bloodstream isolates. Putative plasmids predicted to contain iuc or ybt genes were inferred and clustered using MOB-suite: iuc1 (A), iuc5 and iuc unknown (B), ybt4 (C), and iuc2 (D). Individual nodes represent a plasmid, and different colors represent a different plasmid group. Two plasmids are connected by an edge if their Jaccard index is ≥ 0.95. Networks were graphed using Cytoscape. Hybrid ESBL-virulence or carbapenemase-virulence plasmids are outlined with a dashed line and labeled with the type of beta-lactamase. Networks were graphed using Cytoscape. [file 12879_2022_7558_MOESM12_ESM.pdf]

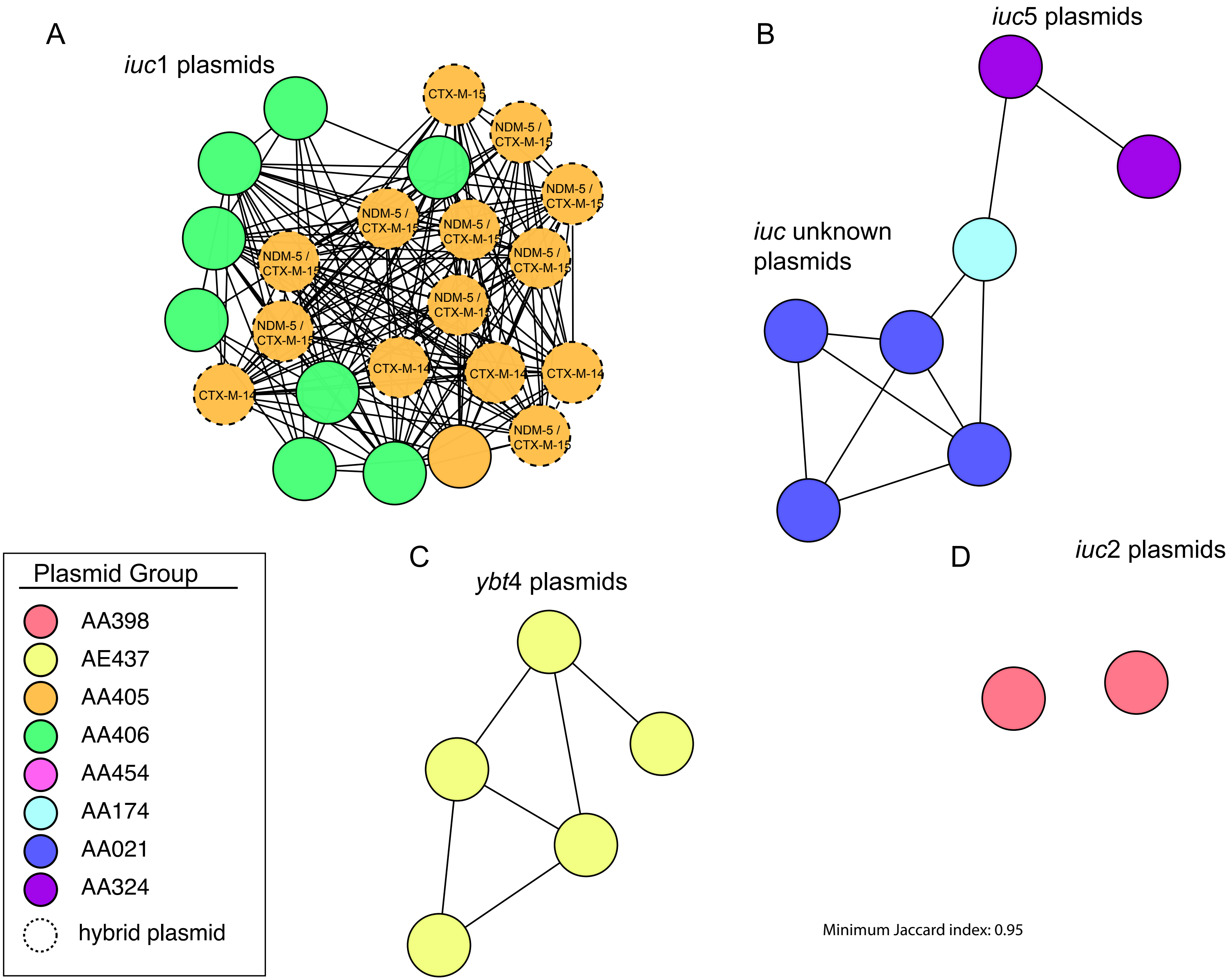
A
iuc1 plasmids
CTX-M-15
NDM-5 / CTX-M-15
CTX-M-14
B
iuc5 plasmids
iuc unknown plasmids
C
ybt4 plasmids
D
iuc2 plasmids
Plasmid Group
AA398
AE437
AA405
AA406
AA454
AA174
AA021
AA324
hybrid plasmid
Minimum Jaccard index: 0.95
